# mRNA export through an additional cap-binding complex consisting of NCBP1 and NCBP3

**DOI:** 10.1038/ncomms9192

**Published:** 2015-09-18

**Authors:** Anna Gebhardt, Matthias Habjan, Christian Benda, Arno Meiler, Darya A. Haas, Marco Y. Hein, Angelika Mann, Matthias Mann, Bianca Habermann, Andreas Pichlmair

**Affiliations:** 1 Innate Immunity Laboratory, Max-Planck Institute of Biochemistry, Martinsried, Munich D-82152, Germany; 2 Department of Structural Cell Biology, Max-Planck Institute of Biochemistry, Martinsried, Munich D-82152, Germany; 3 Department of Proteomics and Signal Transduction, Max-Planck Institute of Biochemistry, Martinsried, Munich D-82152, Germany; 4 Bioinformatics Core Facility, Max-Planck Institute of Biochemistry, Martinsried, Munich D-82152, Germany

## Abstract

The flow of genetic information from DNA to protein requires polymerase-II-transcribed RNA characterized by the presence of a 5′-cap. The cap-binding complex (CBC), consisting of the nuclear cap-binding protein (NCBP) 2 and its adaptor NCBP1, is believed to bind all capped RNA and to be necessary for its processing and intracellular localization. Here we show that NCBP1, but not NCBP2, is required for cell viability and poly(A) RNA export. We identify C17orf85 (here named NCBP3) as a cap-binding protein that together with NCBP1 forms an alternative CBC in higher eukaryotes. NCBP3 binds mRNA, associates with components of the mRNA processing machinery and contributes to poly(A) RNA export. Loss of NCBP3 can be compensated by NCBP2 under steady-state conditions. However, NCBP3 becomes pivotal under stress conditions, such as virus infection. We propose the existence of an alternative CBC involving NCBP1 and NCBP3 that plays a key role in mRNA biogenesis.

## Abstract (web summary)

The processing of RNAs transcribed by RNA polymerase II requires a cap-binding complex (CBC), consisting of NCBP1 and NCBP2. Here, the authors report an alternative CBC formed by NCBP1 and a previously uncharacterized protein, NCBP3 that is critical for RNA processing under cellular stress conditions.

## Graphical abstract

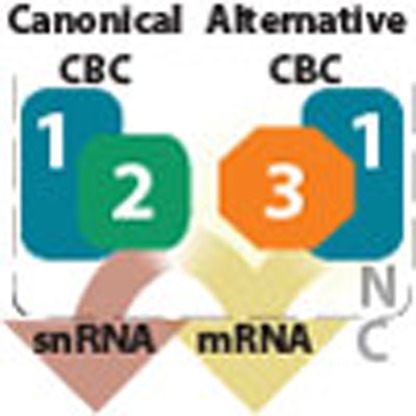

Expression of all germline-encoded genetic information in eukaryotes requires RNA transcription through polymerase complexes and subsequent RNA processing, export and translation. Polymerase II transcripts, such as messenger (mRNA), antisense (asRNA), long intergenic non-coding (lincRNA) and small nuclear RNA (snRNA) are marked by an N7-methylated guanine (m^7^G) ‘cap structure' that is co-transcriptionally attached to the 5′-end of the RNA and serves as a signal for engaging proteins required for downstream processing^1^. Consistent with this notion, splicing and export of snRNA and mRNA can be inhibited by exogenously providing cap analogues^2^,^3^,^4^. The RNA cap structure is bound by the highly conserved nuclear cap-binding complex (CBC), a central factor, known to orchestrate most downstream RNA biogenesis processes such as pre-mRNA splicing, 3′-end processing, nonsense-mediated decay, nuclear–cytoplasmic transport and recruitment of translation factors in the cytoplasm^1^,^5^,^6^,^7^,^8^. The CBC consists of a heterodimer formed by nuclear cap-binding protein 2 (NCBP2, also known as CBP20), which directly associates with the RNA cap, and NCBP1 (also known as CBP80), which stabilizes NCBP2 and serves as an adaptor for other RNA processing factors^9^,^10^,^11^,^12^. The central role of the CBC is demonstrated by short interfering RNA (siRNA)-mediated depletion of NCBP1, which results in deregulated expression of several hundred genes, a reduction in the cell proliferation rate^13^ and reduction of co-transcriptional spliceosome assembly^14^. NCBP1 directly binds the mRNA export factor ALYREF, and ‘CBC competition experiments' using excess of capped RNA led to the conclusion that NCBP1 is involved in mRNA and U snRNA export from the nucleus^15^. However, despite the apparent requirement of NCBP1 for export of capped RNA, antibody-mediated inhibition of NCBP2 *in vivo* only impairs export of U snRNA, but not mRNA^8^. In addition to these data, several genome-wide RNA interference (RNAi)-based screens in human cells that allow assessment of loss-of-function phenotypes in an unbiased manner found that depletion of NCBP1 negatively affects cell growth and viability, whereas depletion of NCBP2 showed only weak phenotypes^16^,^17^,^18^. Collectively, these data suggest that the two CBC subunits only in part share the same biological function. Thus, we questioned whether an additional protein exists that has partially redundant activity to NCBP2 and associates with NCBP1 to form an alternative CBC.

Here, we identify the largely uncharacterized protein C17orf85 (NCBP3) as a novel genuine cap-binding protein that directly interacts with NCBP1 and binds cellular mRNA. Similar to NCBP2, NCBP3 is non-essential under steady-state conditions. However, simultaneous depletion of NCBP2 and -3 mimics the phenotype of NCBP1 knockdown. Notably, NCBP3 becomes pivotal under cellular stress conditions, such as virus infections. We propose the existence of a canonical and an alternative CBC that is fundamental for mRNA biogenesis of higher eukaryotes.

## Results

### Loss of NCBP1 and NCBP2 leads to different phenotypes

**Figure 1:**
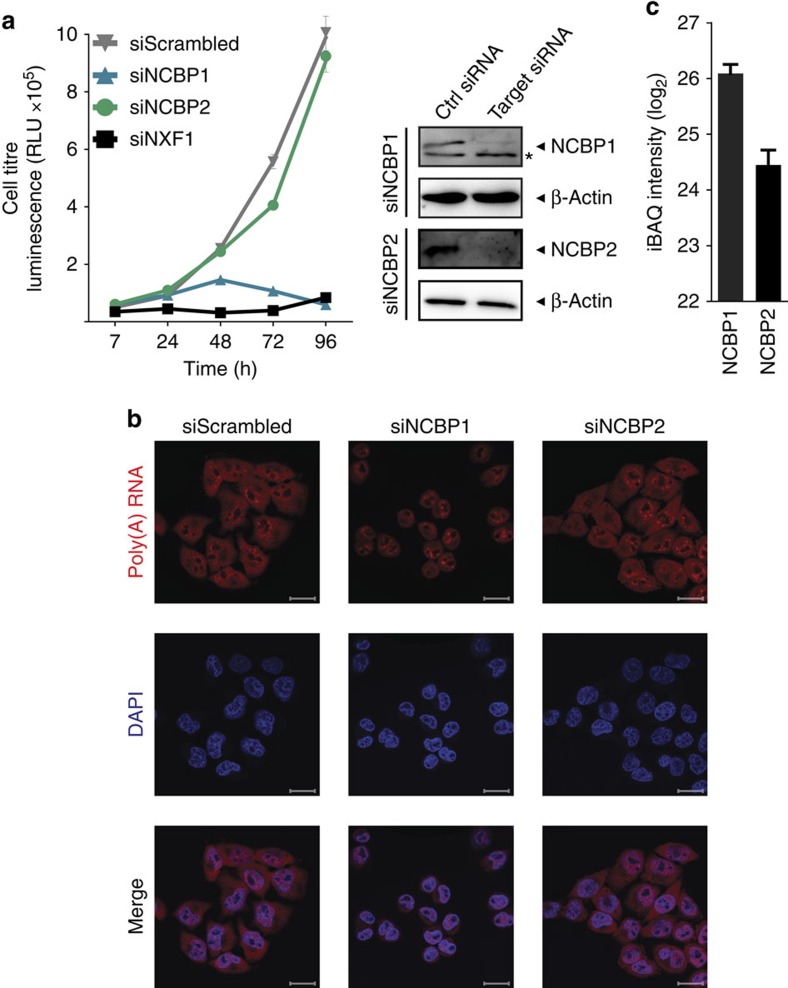
Cell growth and poly(A) RNA distribution after knockdown of NCBP1 or NCBP2. (**a**) Growth of HeLa cells after RNAi-mediated knockdown (left panel). Cells were treated twice with siRNAs against NCBP1, NCBP2, NXF1 or non-targeting siRNA control (siScrambled), and cell titre determined by a luminescence-based cell viability assay at the indicated time points. The graph shows the mean±s.d. of two individual treatments measured in triplicates. One representative experiment of three is shown. RLU, relative light units. Knockdown efficiency of NCBP1 and NCBP2 was confirmed by western blotting against indicated proteins (right panel). Asterisk: nonspecific band. (**b**) Poly(A) RNA distribution in HeLa cells 48 h after repeated RNAi-mediated knockdown. HeLa cells were transfected with the indicated siRNAs, and localization of poly(A) RNA (red) was stained by RNA fluorescence *in situ* hybridization (RNA-FISH) using fluorescently labelled oligo (dT) as probe. DAPI (blue) was used to visualize nuclei. Shown confocal images are representative for three independent experiments. Scale bar, 20 μm. (**c**) Abundance of NCBP1 and NCBP2 in HeLa cells. Complete HeLa cell lysates were analysed by LC–MS/MS. Shown are average iBAQ intensities for NCBP1 and NCBP2 of 11 measurements±s.d.

To study the individual requirement of CBC components NCBP1 and NCBP2 for cell viability, we evaluated cell growth after their transient siRNA-mediated depletion in HeLa cells. As expected, depletion of NCBP1 or the Nuclear RNA export factor 1 (NXF1, also known as TAP) severely affected cell growth (Fig. 1a). Surprisingly, after selective depletion of NCBP2, we did not observe a similar effect on cell viability. Likewise, depletion of NCBP2 did not affect intracellular distribution of poly-adenylated (poly(A)) mRNA, as tested by RNA fluorescence *in situ* hybridization (RNA-FISH) (Fig. 1b). In contrast, loss of NCBP1 resulted in accumulation of poly(A) RNA in the nucleus, confirming a critical role of NCBP1 in mRNA export^15^. Since NCBP1 cannot directly associate with capped RNA, we hypothesized on the existence of a protein with a redundant function to NCBP2, that is, a protein with the ability to bridge the association between capped RNA and NCBP1. Contribution of NCBP1 to additional protein complexes is supported by protein expression data based on quantitative mass spectrometry (MS) that suggest about three times higher abundance of NCBP1 as compared with NCBP2 (Fig. 1c).

### Identification of C17orf85 as cap-binding protein NCBP3

**Figure 2:**
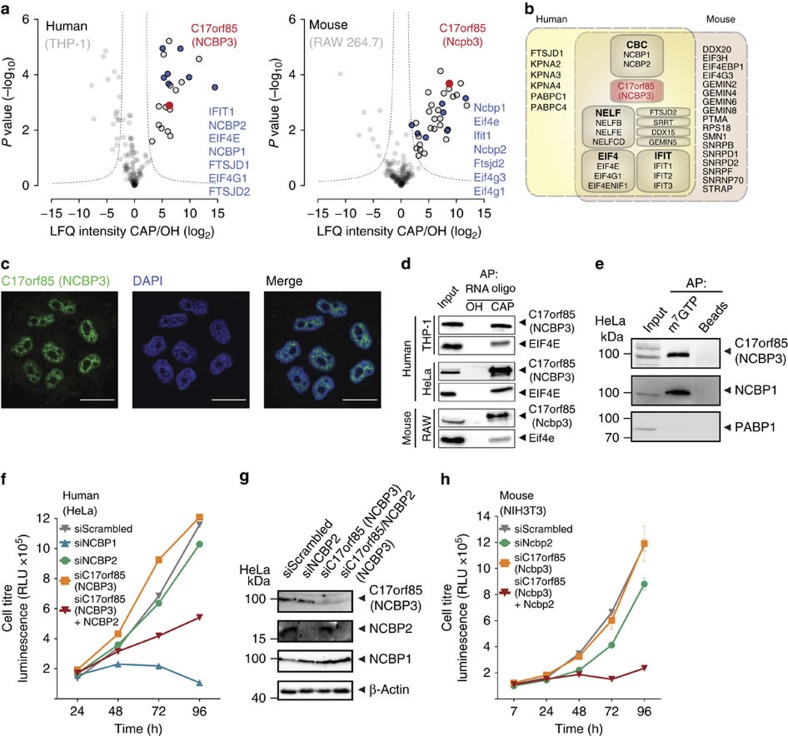
C17orf85/NCBP3 binds capped RNA and compensates for loss of NCBP2. (**a**) Proteins enriched with 5′-capped (CAP) and uncapped (OH) RNA from THP-1 (human, left panel) and RAW 264.7 (mouse, right panel) macrophages were analysed by LC–MS/MS. Volcano plots show the average degrees of enrichment by CAP- over OH-RNA (ratio of label-free quantification (LFQ) protein intensities; *x* axis) and *P* values (*t*-test; *y* axis) for each protein. Significantly enriched proteins (circled in black) are separated from background proteins by a hyperbolic curve (dotted line). C17orf85/NCBP3 (red dot) and proteins known to bind capped RNA (blue dots) are highlighted. Three independent affinity purifications were performed for both baits. (**b**) Shared enrichment network. Proteins significantly enriched by CAP-RNA from either human or mouse cells (data from Fig. 1a). Proteins belonging to known protein complexes were grouped into boxes (CBC, cap-binding complex; NELF, negative elongation factor; IFIT, interferon-induced proteins with tetratricopeptide repeats; EIF4, eukaryotic translation initiation factor 4). (**c**) Subcellular localization of endogenous NCBP3 assessed by confocal microscopy. HeLa cells were grown on glass coverslips and stained for NCBP3 (green) and DAPI (blue). Scale bar, 20 μM. (**d**) Binding of endogenous C17orf85/NCBP3 to synthetic RNA oligomers (RNA oligo). Western blot analysis of precipitates after affinity purification (AP) with hydroxylated (OH) and m^7^G-capped (CAP) RNA oligos from murine RAW 264.7, human THP-1 and HeLa cells using antibodies against C17orf85/NCBP3 and the cap-binding control protein EIF4E. (**e**) Western blot analysis of m^7^GTP-precipitated proteins from HeLa cells detecting C17orf85/NCBP3, NCBP1 and PABP1 as control. (**f**,**h**) Growth of HeLa (**f**) and NIH3T3 (**h**) cells after RNAi-mediated knockdown. Cells were treated twice with siRNAs targeting NCBP2 or NCBP3 alone, NCBP3 together with NCBP2, or nonspecific siRNA (siScrambled) as control. The cell titre was determined by a luminescence-based cell viability assay at the indicated time points. The graph shows the mean±s.d. of two individual treatments measured in triplicates. One representative experiment of three is shown. RLU, relative light units. (**g**) Knockdown efficiency and specificity of NCBP2 and NCBP3 in HeLa cells was confirmed by western blotting against indicated proteins.

A protein with redundant function to NCBP2 should have the ability to associate with the RNA cap structure and to bind to NCBP1 to engage factors required for RNA biogenesis. To identify potential candidate proteins, we performed affinity purification followed by liquid chromatography tandem MS (AP–LC–MS/MS) using lysates from human and mouse cell lines and capped and non-capped RNA as baits (Supplementary Fig. 1a). Capped RNA reliably enriched proteins known to associate directly with the RNA cap structure, among them the CBC (consisting of NCBP1 and -2)^6^, the genuine cap-binding protein EIF4E^1^ and the antiviral protein IFIT1 (ref. 19) from human THP-1 and RAW 264.7 cell lysates (Fig. 2a,b; Supplementary Fig. 1; Supplementary Data 1). Among the proteins identified in human and murine cell lysates was only one protein, C17orf85 (also known as ELG), for which we could not explain its association with RNA in a cap-dependent manner^20^,^21^. C17orf85 is a highly conserved protein, and orthologues can be found in the fungal and metazoan kingdoms (Supplementary Fig. 2; Supplementary Data 2), suggesting evolutionary conservation for more than one billion years. However, based on currently available whole-genome sequencing data, it seems to have been lost at least by some *Muscomorpha* and *Saccharomycetaceae*, which include *Drosophila melanogaster* and *Saccharomyces cerevisiae*, respectively. Recent data on distribution of proteins in human tissues suggest that C17orf85 is expressed in all tissues^22^. Consistent with a potential redundant role to NCBP2, endogenous and green fluorescent protein (GFP)-tagged C17orf85 showed predominantly nuclear localization (Fig. 2c; Supplementary Fig. 3a). Subcellular fractionation confirmed the nuclear localization, but also identified a cytoplasmic proportion (Supplementary Fig. 3b), a phenotype often found for proteins involved in RNA export^23^,^24^. Here, we name C17orf85 NCBP3 for nuclear cap-binding protein 3.

To confirm cap-dependent binding of NCBP3, we used capped and non-capped RNA as bait for AP, followed by western blotting. NCBP3 precipitated with capped RNA in human THP-1 and murine RAW 264.7 cells comparably well as the cap-binding protein EIF4E (Fig. 2d). The canonical CBC has been mainly studied in human HeLa cells. We therefore confirmed the presence and binding of NCBP3 to capped RNA in HeLa cells (Fig. 2d). In line with specific affinity for the cap structure, NCBP3 was precipitated from cell lysates using N7-methylated guanosine-5′-triphosphate (m^7^GTP)-coupled beads (Fig. 2e). m^7^GTP also precipitated the CBC component NCBP1 but not poly-A-binding protein 1 (PABP1), confirming specificity of this assay.

To functionally assess the potential contribution of NCBP3 to compensate for loss of NCBP2, we employed siRNA-based knockdown and tested for cell growth in HeLa cells. Depletion of either NCBP2 or NCBP3 alone did not considerably affect cell growth (Fig. 2f) despite efficient and specific knockdown as tested by quantitative reverse transcription (qRT)–PCR, western blotting and quantitative proteomics (Fig. 2g; Supplementary Fig. 4; Supplementary Data 3). Although we cannot rule out that residual amounts of the proteins are sufficient for biological activity, simultaneous depletion of NCBP2 and NCBP3 with comparable efficiency markedly reduced cell growth and mimicked loss of NCBP1 (Fig. 2f,g; Supplementary Fig. 4c; Supplementary Data 3). An analogous experiment in murine NIH3T3 cells confirmed that depletion of both, Ncbp2 and Ncbp3 also inhibited cell growth in mouse cells, whereas individual knockdown of the two proteins did not (Fig. 2h). In summary, these experiments suggest that loss of NCBP2 and NCBP3 is synthetically lethal, and highlight NCBP3 as a prime candidate for formation of an alternative CBC in mammalian cells.

### Mechanism of cap-RNA binding by NCBP3

**Figure 3:**
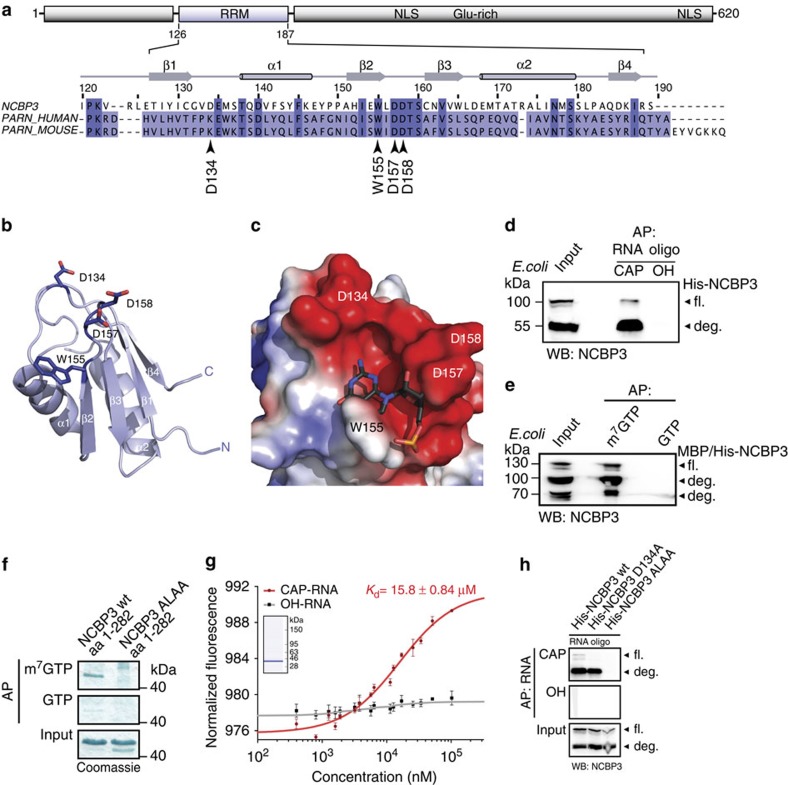
Biochemical and biophysical properties of the cap-binding region of NCBP3. (**a**) Top, predicted features of NCBP3 and their positions in the full-length protein: RRM fold (aa 126–187, highlighted in blue), nuclear localization signal (NLS) and glutamine-rich region (Glu-rich). Bottom, secondary structure elements of NCBP3 (aa 120–189) and sequence alignment with human and mouse PARN RRMs. Arrowheads show residues chosen for a mutational analysis. (**b**) Homology model of the RRM of NCBP3. Secondary structure elements of the predicted RRM fold and residues chosen for mutational analysis are annotated. (**c**) Modelled RRM domain of NCBP3 superimposed with mouse PARN bound to m7GpppG (for clarity only the m^7^Gp moiety is shown, PDB code 3d45) to indicate the putative binding site. The model shows the solvent-accessible surface coloured according to electrostatic charge distribution. (**d**) Binding of *E. coli*-expressed NCBP3 to RNA oligonucleotides. Western blot after affinity purification with 5′-CAP- or OH-RNA using lysate from bacteria expressing recombinant full-length NCBP3. Full-length (fl.) recombinant NCBP3 and N-terminal degradation products (deg.) are indicated. (**e**) m^7^GTP versus GTP-affinity purification and western blotting of recombinant NCBP3 from *E. coli* lysates. (**f**) Coomassie-stained SDS–polyacrylamide gel electrophoresis gel after affinity purification of purified wild-type and mutant (ALAA) NCBP3 (aa 1–282) using m^7^GTP and GTP as bait. (**g**) Binding affinity of NCBP3 (aa 1–282) to 5′-CAP- or 5′-OH-RNA as determined by microscale thermophoresis (MST). The graph shows normalized fluorescence versus concentration of NCBP3. Mean±s.d. of three independent measurements are shown. The inset shows the quality of the recombinant NCBP3 (aa 1–282) as analysed by an Agilent protein chip. (**h**) Binding of recombinant wild-type and mutant NCBP3 to 5′-capped RNA. Western blot after affinity purification with biotinylated RNA oligos harbouring either a 5′-CAP or OH structure using lysate from *E. coli* expressing either recombinant full-length wild-type NCBP3 (wt), NCBP3 where aspartic acid at position 134 had been mutated to alanine (D134A), or where tryptophan at position 155 and two aspartic acids at position 157 and 158, respectively, had been mutated to alanines (ALAA).

NCBP1 lacks a canonical RNA or cap recognition domain. Hence, binding to capped RNA is mediated by its partner NCBP2. As suggested above, in an alternative scenario, cap binding could also be mediated by NCBP3. Indeed, homology-based structure prediction of NCBP3 suggested the presence of a canonical RNA recognition motif (RRM) fold (β1–α1–β2–β3–α2–β4)^25^ in the N-terminal region of the protein (residues 126–187) (Fig. 3a). The C-terminal part of NCBP3 is unstructured, and a homology search did not reveal the presence of any other predicted domains. Two nuclear localization signals explain the predominant nuclear staining in confocal microscopy and subcellular fractionation analysis (Fig. 2c; Supplementary Fig. 3). Comparative modelling^26^ using HHpred resulted in a structural model for the core RRM domain (Fig. 3b) based on the RRM of poly(A)-specific ribonuclease PARN, a protein known to bind the cap analogue m^7^GpppG. The RRM of NCBP3 shares high sequence homology (identity of 21.4% for residues 121–191) and critical residues with the RRM of PARN (Fig. 3a–c; Supplementary Fig. 5a)^27^,^28^,^29^ as well as with the RRM of NCBP2, although with lower sequence identity (16.4%) (Supplementary Fig. 5b). To study whether NCBP3 has the ability to directly bind capped RNA, we generated recombinant NCBP3 in *Escherichia coli*. Both, the full-length protein and an N-terminal cleavage fragment of NCBP3 precipitated with RNA in a cap-dependent manner (Fig. 3d). The hallmark of the cap structure is a methyl group at the N7 position of the guanosine, and genuine cap-binding proteins such as EIF4E have the ability to distinguish between N7-methylated and unmethylated GTP. Consistent with this notion, recombinant NCBP3 selectively bound m^7^GTP but not GTP (Fig. 3e). A highly purified N-terminal fragment (position 1–282 amino acids) consisting of the core RRM was sufficient for selective binding (Fig. 3f). Microscale thermophoresis (MST)-based affinity measurements showed that the RRM-containing fragment has an affinity to cap-RNA in the low micromolar range (15.8±0.84 μM), which is comparable to the affinity of PARN to m^7^GpppG^28^,^30^ (Fig. 3g).

In case of PARN, a conserved WXDD motif located in loop β2–β3 is involved in m^7^GpppG binding. N7-methylated GTP is bound to a surface pocket on the C-terminal RRM of PARN mainly through π-interaction with tryptophan W468 and polar interactions with two aspartic acids, D471 and D470 (ref. 29). According to the predicted model, NCBP3 has a similar motif (WLDD) forming a negatively charged groove (D157, D158) with an aromatic platform for π-interaction (W155; Fig. 3b,c). To test whether NCBP3 uses these residues for cap-RNA binding, we generated point mutants in this putative RNA-binding motif. Whereas a single D134A mutation in close vicinity of the RNA-binding groove only marginally affected cap binding, mutation of the NCBP3 WLDD motif to ALAA resulted in loss of binding to capped RNA and m^7^GTP (Fig. 3f,h; Supplementary Fig. 6a). To ensure that the introduced mutations did not influence the overall integrity and folding of the recombinant proteins, we used circular dichroism (CD) spectroscopy to analyse the secondary structure content. Both samples showed comparable CD spectra, irrespective of the introduced modification, suggesting that both proteins adopt a similar fold (Supplementary Fig. 5b). This shows that NCBP3 binds to the cap structure through a canonical binding mechanism using a core RRM and the conserved WLDD motif.

### NCBP3 associates with NCBP1 and mRNA processing factors

**Figure 4:**
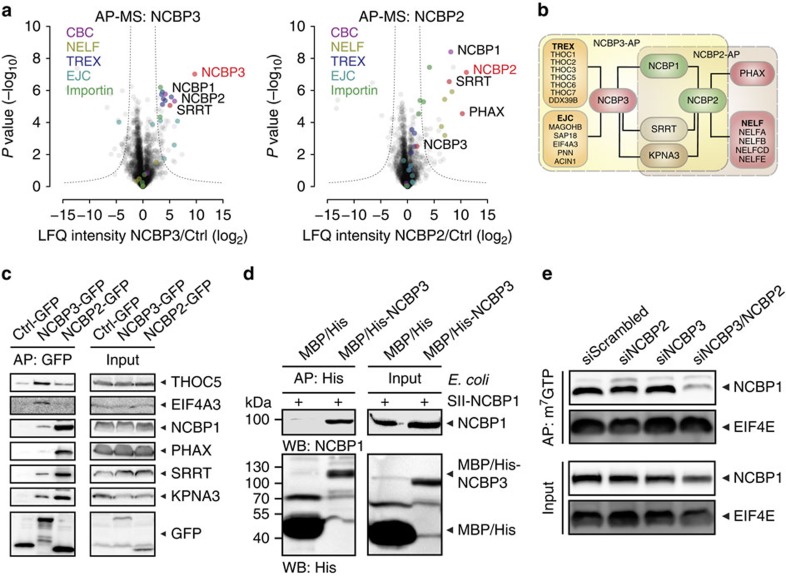
Association of NCBP3 and NCBP2 with NCBP1 and proteins involved in RNA processing. (**a**) GFP-tagged NCBP3, NCBP2 or control protein (ctrl) stably expressed in HeLa cells were precipitated and associated proteins were analysed by LC–MS/MS. Volcano plots show the average degrees of enrichment by NCBP3 or NCBP2 over ctrl (ratio of LFQ protein intensities; *x* axis) and *P* values (*t*-test; *y* axis) for each protein. Bait proteins (red letters), proteins of the exon junction complex (EJC, turquoise dots) or the TREX complex (purple dots), CBC (magenta dots), NELF (olive dots), importin (green dots) and NCBP3, SRRT and PHAX (red dots) are highlighted. Significantly enriched proteins are separated from the background by a hyperbolic curve. Four independent affinity purifications were performed for each bait. (**b**) Schematic illustration of AP–MS analysis in **a** focusing on proteins involved in RNA biogenesis. (**c**) Western blot analysis of representative GFP-precipitated proteins identified by AP–MS analysis. (**d**) Binding of NCBP1 to NCBP3 in *E. coli*. Western blot after His-precipitation using RNAse-treated lysates from bacteria co-expressing recombinant SII-tagged NCBP1 and full-length MBP/His-tagged NCBP3 or MBP/His as control. (**e**) NCBP2 and NCBP3 are required for NCBP1 association to m^7^GTP. Lysates of HeLa cells treated twice with siRNA against NCBP2, NCBP3 or both were used for m^7^GTP-affinity purification (m^7^GTP-AP), followed by western blot analysis. The m^7^GTP-binding protein EIF4E served as control.

To gain further insights into biological functions of NCBP3, we performed quantitative shotgun AP–LC–MS/MS (Supplementary Fig. 7a)^31^. To this aim, we used HeLa cell lines that stably express GFP-tagged NCBP3, NCBP2 and an unrelated control protein (RAB5C) from their endogenous promoters as baits^32^,^33^. NCBP3 significantly enriched for 88 proteins (Fig. 4a; Supplementary Fig. 7b; Supplementary Data 4) that are mostly related to RNA biogenesis, particularly mRNA transport^1^,^5^,^6^,^7^,^34^,^35^ (Supplementary Fig. 7c). Most importantly, the CBC component NCBP1 was found to interact with NCBP3 (Fig. 4a,b; Supplementary Fig. 7b). Besides NCBP1, NCBP3 prominently interacted with components of the TRanscription EXport (TREX) complex (for example, THOC1, -2, -3, -5, -6, -7, DDX39B) that function in mRNA export from the nucleus. NCBP3 further associated with proteins belonging to the exon junction complex (EJC) (MAGOHB, SAP18, EIF4A3, PNN and ACIN1), which are deposited on spliced mRNAs and are involved in mRNA stability. Although NCBP2 precipitates also contained components of the TREX complex and EJC, their enrichment was not statistically significant as compared with control APs (Fig. 4a; Supplementary Fig. 7b). However, NCBP2 precipitates showed high enrichment for the phosphorylated adaptor for RNA export (PHAX) known for its function in U snRNA nuclear export^36^ and the negative elongation factor (NELF) complex (consisting of NELFA, NELFB, NELFCD and NELFE), which participates in 3′-end processing of histone mRNAs^13^ (Fig. 4a,b; Supplementary Fig. 7b). We independently confirmed the AP–LC–MS/MS data by co-precipitation experiments, followed by western blotting (Fig. 4c). We concluded that NCBP2 and NCBP3 can both bind NCBP1. However, directly comparing NCBP3 and NCBP2 suggests that they preferentially bind proteins involved in mRNA processing/export and snRNA export, respectively.

### An alternative CBC

The association of NCBP3 to NCBP1 suggested formation of an alternative CBC. To formally test whether NCBP3 and NCBP1 directly bind to each other, we co-expressed both proteins in *E. coli* and performed co-precipitation experiments. In line with our hypothesis, NCBP1 co-precipitated with NCBP3 but not with a negative control, endorsing a direct interaction between NCBP3 and NCBP1 (Fig. 4d) and suggesting the formation of an alternative CBC that is reminiscent of the canonical CBC consisting of NCBP1 and -2. This result further suggested that NCBP2 and NCBP3 have the ability to individually bridge the association of NCBP1 to capped RNA. To test this in a mammalian system, we depleted NCBP2, NCBP3 or both proteins in HeLa cells and precipitated NCBP1 using m^7^GTP beads. Consistent with the presence of two CBCs, NCBP1 could be recovered from m^7^GTP beads in the absence of either NCBP2 or NCBP3 (Fig. 4e). However, in the absence of NCBP2 and -3, NCBP1 binding to m^7^GTP beads was markedly impaired, indicating that in mammalian cells NCBP1 requires either NCBP2 or NCBP3 for cap-RNA association. This effect was specific for NCBP1, since the cap-binding protein EIF4E precipitated with m^7^GTP beads with similar efficiency in all cases.

Binding of NCBP2 to NCBP1 requires residues located in the RRM. Sequence alignments of the NCBP2 and NCBP3 RRM domains showed that the residues critical for NCBP1 binding in NCBP2 are not conserved in NCBP3 (Supplementary Fig. 5b). Consistent with this finding, yeast two-hybrid data indicated that NCBP3 binds NCBP1 through its unstructured C-terminal region^37^. These findings provide evidence that NCBP2 and NCBP3 use different domains to bridge the association of NCBP1 to capped RNA.

### Specificity of RNA types bound by NCBP2 and NCBP3

**Figure 5:**
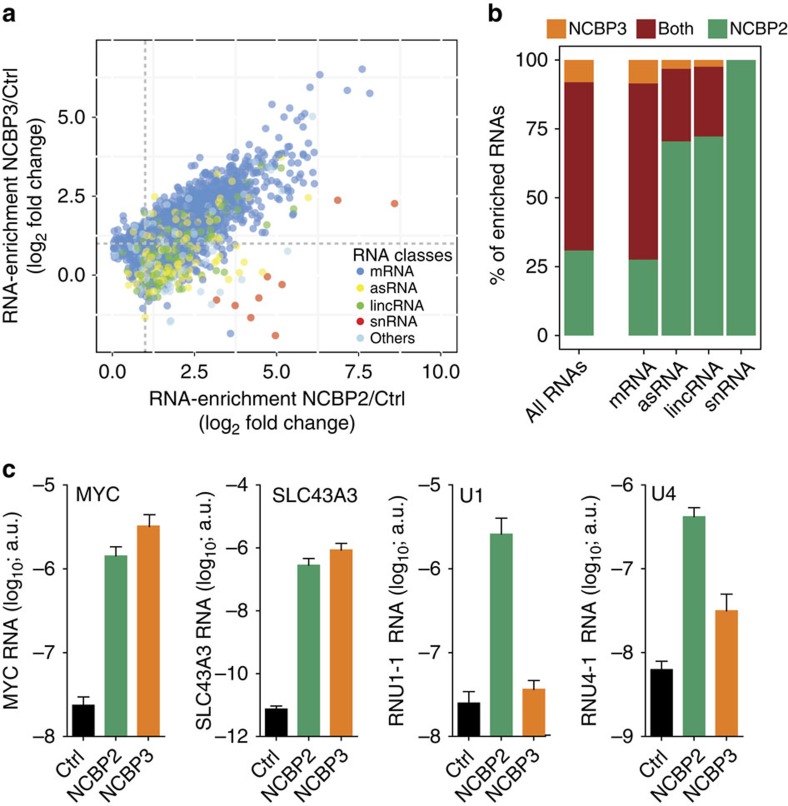
Global analysis of NCBP3- and NCBP2-bound RNAs. Binding of endogenous RNA to NCBP3 and NCBP2. GFP-tagged NCBP3, NCBP2 or control protein (ctrl) stably expressed in HeLa cells were precipitated and associated RNAs were analysed by deep sequencing on the Illumina HiSeq platform (**a**,**b**) or qRT–PCR (**c**). All affinity purifications were performed in triplicates. (**a**) Scatter plot showing enrichment (FPKM_bait_+2/FPKM_control_+2) of transcripts binding to NCBP2 (*x* axis) and NCBP3 (*y* axis) quantified on the gene level. RNAs are colour-coded according to their annotated RNA types in Ensembl. (**b**) Percentage of enriched RNA types binding to NCBP3, NCBP2 or both proteins. RNAs have been considered enriched at a fold change bait over control >2, a false discovery rate (FDR) <0.01 and a minimal read count of 10. (**c**) Validation of sequencing data by qRT–PCR. RNA in NCBP2, NCBP3 or control (ctrl) precipitates was amplified by qRT–PCR using specific primers for two mRNAs (MYC and SLC43A3) and two snRNAs (U1 and U4). Data represent the mean±s.d. a.u., arbitrary units.

Collectively, our data show that NCBP3 has affinity for the RNA cap structure and bridges NCBP1 binding to m^7^GTP in an NCBP2-independent manner. The binding of different RNA processing proteins highlights the possibility that distinct types of RNA associate with either NCBP2 or NCBP3. To test this, we precipitated GFP-tagged NCBP3, NCBP2 or control and sequenced bound RNA by deep sequencing (RNA immunoprecipitation followed by deep sequencing, RIP-Seq). In agreement with the ability of NCBP3 and NCBP2 to associate with the cap structure, both proteins enriched for capped RNAs as compared with control precipitates (Supplementary Data 5). Plotting the individual RNAs enriched by NCBP2 and NCBP3 and colour coding for the respective RNA species revealed an RNA-binding pattern for both proteins: NCBP2 precipitates showed particularly high enrichment for snRNA, lincRNA and asRNA (Fig. 5a,b). Among individual mRNAs, histone mRNAs were mainly apparent in NCBP2 precipitates (Supplementary Data 5). In contrast, NCBP3 associated with mRNA, but did not bind snRNA, and comparably less asRNA and lincRNA (Fig. 5b). The majority of mRNAs bound both NCBP2 and -3 with only a limited number of mRNAs showing more than twofold enrichment in either NCBP3 versus NCBP2 (Fig. 5b, orange) or NCBP2 versus NCBP3 precipitates (Fig. 5b, green). We validated the differences in binding to mRNA and snRNA using qRT–PCR (Fig. 5c). NCBP2 bound mRNAs (MYC, SLC43A3) and snRNAs (U1, U4), whereas NCBP3 selectively bound mRNAs (MYC, SLC43A3) but did not enrich for snRNAs. Collectively, this suggested that snRNA, lincRNA and asRNA preferentially bind NCBP2, while mRNAs can be bound by either NCBP2 or -3. The RIP-Seq data were in strong agreement with the protein–protein interaction data (Fig. 4) that showed selective binding of PHAX (transporting snRNA) and NELF (3′-end processing of replication-dependent histone mRNAs) to NCBP2 (refs 5, 13).

### NCBP2 and -3 individually contribute to mRNA export

**Figure 6:**
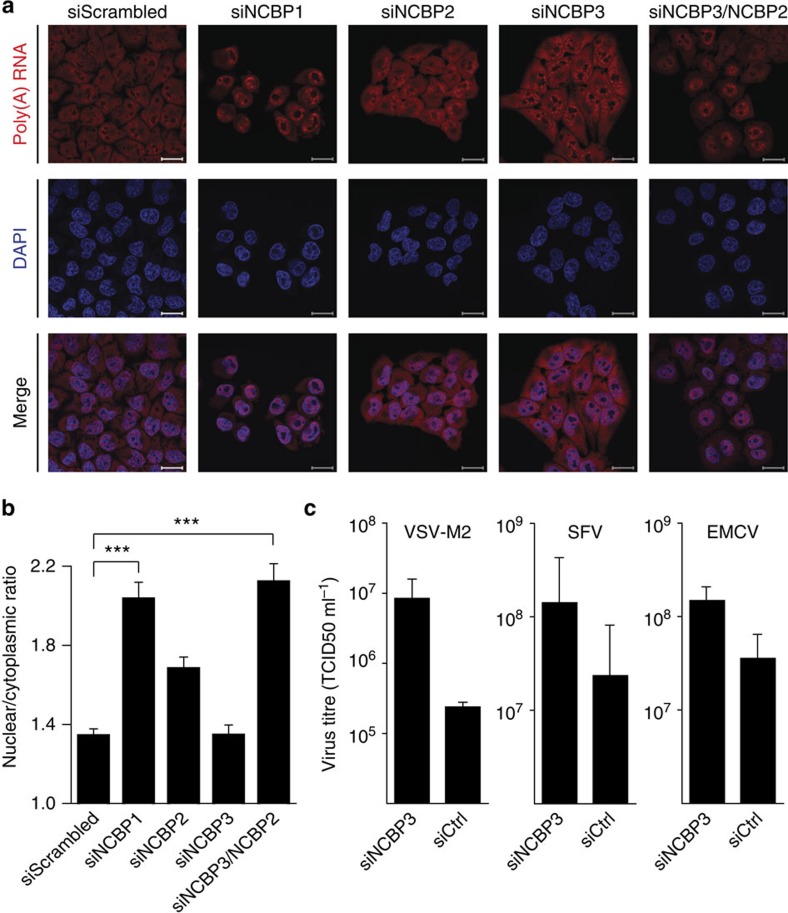
Functional analysis of NCBP3 at normal and virus-challenged growth conditions. (**a**,**b**) Poly(A) RNA distribution in HeLa cells 72 h after RNAi-mediated knockdown. HeLa cells were transfected with the indicated siRNAs and localization of poly(A) RNA (red) was detected by fluorescence *in situ* hybridization (FISH) using fluorescently labelled oligo (dT) as probe. DAPI (blue) was used to visualize nuclei. (**a**) Representative images of poly(A) RNA distribution after RNAi-mediated knockdown. Scale bar, 20 μm. (**b**) The nuclear to cytoplasmic ratio of poly(A) RNA intensity was determined for a minimum of 125 cells per condition. Data represent the average ratio of the nuclear/cytoplasmic poly(A) RNA signal±s.d. ***P<0.001 as analysed by one-way analysis of variance statistics with Tukey's post-test. (**c**) Virus growth in HeLa cells after RNAi-mediated knockdown. HeLa cells were treated with siRNA against NCBP3 or nonspecific siRNA (siGFP) as control and infected with mutant vesicular stomatitis virus (VSV-M2; M51R substitution in the matrix protein), Semliki Forest virus (SFV) or encephalomyocarditis virus (EMCV). Virus titres in supernatants were determined by TCID50 24 h post infection.

Our data suggested that both, NCBP2 and -3 have the ability to bind mRNA and to recruit proteins involved in RNA processing. We therefore tested whether both proteins individually contribute to nuclear–cytoplasmic transport of mRNA. In RNA-FISH experiments, depletion of either NCBP2 or -3 did not considerably change poly(A) RNA distribution (Fig. 6a). Remarkably, simultaneous depletion of NCBP2 and -3 trapped poly(A) RNA in the nucleus. We quantified the ratio between the nuclear and cytoplasmic poly(A) RNA signal and found similar ratios for co-depletion of NCBP2 and -3 as compared with depletion of NCBP1 (Fig. 6b). To assess whether this is a direct effect of individual NCBP depletion or an indirect effect on expression of mRNA processing factors, we performed quantitative proteomics of siRNA-treated cells, which allows simultaneous quantification of over 5,000 proteins (Supplementary Data 3). As expected, NCBP1 was required for stability of NCBP2 (ref. 10); however, we could not observe an influence on NCBP1 and -2 levels after NCBP3 knockdown and vice versa (Supplementary Fig. 4c). The abundance of mRNA processing factors such as EJC, TREX and NELF components remained similarly unchanged (Supplementary Fig. 4c), collectively emphasizing a direct effect of NCBP depletion on poly(A) RNA export. Our data thus indicate that mRNA can be exported through the activity of either the canonical CBC consisting of NCBP1/-2 or the alternative CBC formed by NCBP1/-3. Furthermore, these data suggest that both CBCs can have functionally redundant activities under steady-state conditions.

### NCBP3 activity is critical to inhibit virus growth

Perturbation of the RNA export machinery components often shows loss-of-function phenotypes under challenging conditions^38^. Infection with pathogens induces cellular stress and requires swift expression, processing and export of RNAs coding for antiviral proteins or factors required for virus growth^39^,^40^. We therefore tested the effect of NCBP3 knockdown on growth of viruses that replicate in the cytoplasm and are not dependent on the cellular mRNA export machinery. HeLa cells treated with siRNA against NCBP3 showed an almost 100-fold increase in growth of vesicular stomatitis virus variant M2 (VSV-M2) (Fig. 6c). Similarly, growth of two other cytoplasmic viruses, Semliki forest virus (SFV) and encephalomyocarditis virus (EMCV), was increased in the absence of NCBP3. These data suggest that NCBP3 becomes fundamental for cellular functions under stress conditions such as virus infection.

## Discussion

The canonical CBC consisting of NCBP1 and -2 is considered to be one of the most fundamental parts of the biogenesis machinery of all capped RNAs. This notion is based on the involvement of the CBC in pre-mRNA splicing, 3′-end processing, intranuclear transport, nuclear–cytoplasmic transport of RNA and nonsense-mediated decay and translation^10^,^14^,^41^. Exogenously added cap analogues impair RNA metabolism, underlining the major importance of the cap structure in this process. However, *in vivo* antibody-mediated inhibition of NCBP2 only affected nuclear export of U snRNAs but not general export of mRNA^8^. Using AP with synthetic capped RNA and unbiased protein–protein interaction studies, we identified the largely uncharacterized protein NCBP3 as a novel genuine cap-binding protein that links NCBP1 to capped RNA, and propose that NCBP1/-3 forms an alternative CBC that is involved in mRNA biogenesis.

Structure-guided binding studies using recombinant NCBP3 clearly established direct cap-binding through a canonical RRM bearing a WLDD motif at the centre of the RNA-binding grove. This motif is also conserved in the canonical cap-RNA-binding protein PARN and mediates affinity to cap-RNA in the low micromolar range. In addition, NCBP3 directly binds NCBP1 when co-expressed in bacteria and thus allows formation of a protein complex with affinity to capped RNA. A direct interaction between NCBP1 and -3 is further suggested from large-scale yeast two-hybrid interaction data^37^. Notably, NCBP2 and -3 were the only two proteins in this screen showing interaction with NCBP1 when being used either as bait or as prey. However, NCBP2 and -3 do not show an apparent homology that would suggest a shared binding mechanism to NCBP1. Both, NCBP2 and -3 have the ability to independently serve as adaptor proteins linking the RNA cap to NCBP1, and only loss of both proteins in HeLa cells reduced NCBP1 association with m^7^GTP. Co-depletion of NCBP2 and -3 is synthetically lethal in human und murine fibroblasts, suggesting redundancy between both proteins. This redundancy can be explained by RIP-seq experiments, showing that NCBP2 and -3 share affinity for the majority of mRNA. In addition to mRNA binding, NCBP2 but not NCBP3 has the ability to bind snRNA. Consistent with the selective affinity for snRNA, only NCBP2 binds the snRNA transport protein PHAX. Our results consolidate reports that are incompatible with the current model of a single CBC for RNA export: while inhibition of NCBP2 selectively impairs U snRNA nuclear–cytoplasmic export, but not of mRNA^9^, NCBP1 is strictly required as an adaptor for mRNA export^15^.

It is commonly accepted that EJC components, the TREX complex, PHAX and the NELF complex are recruited to RNAs by virtue of the CBC. We here show that EJC and TREX precipitate superior with NCBP3 as compared with NCBP2, whereas PHAX and the NELF complex are exclusively enriched in NCBP2 precipitates. It is tempting to speculate that binding of either NCBP2 or -3 induces a conformational change of NCBP1 that in turn allows differential binding of adaptor proteins. Structural analysis of NCBP1 in the context of NCBP2 and -3 bound to capped RNA, as well as binding assays with purified proteins will be necessary to study this in detail.

Given the association with proteins of the EJC and TREX complex, NCBP3 may play a primary role in biogenesis of spliced mRNA. Such a role is supported by genetic correlation: although NCBP3 is remarkably conserved during evolution from fungi to humans, we could not find homologues for NCBP3 in *Saccharomyces cervisiae*. Notably, in *S. cervisiae* only 3% of genes contain introns and only six genes contain two introns^42^. In contrast, the closely related *Schizosaccharomyces pombe* expresses an NCBP3 homologue and codes for introns in 43% of its genes^43^. Loss of NCBP3 in *S. cerevisiae* may therefore be a result of evolutionary adaptation.

The canonical CBC (consisting of NCBP1/-2) guides mRNA from the nucleus into the cytoplasm. Similarly, NCBP3 localizes to the nucleus and partially to the cytoplasm, suggesting that the alternative CBC drives mRNA export to the cytoplasm. RNA bound to the canonical CBC or EIF4E is sensitive to the nonsense-mediated mRNA decay (NMD) pathway^44^,^45^,^46^. Likewise, NCBP1/-3 associated with exported mRNA may be a target of NMD, particularly since the EJC, which co-purified with NCBP3, is known to promote NMD.

Given that RNA metabolism is of central importance for all physiological and pathophysiological processes, we envisage a central role of the alternative CBC in various diseases, as known for many proteins involved in RNA splicing, 3′-end processing and degradation^2^,^41^,^47^. A reason for metazoans to evolve an alternative CBC could be the availability of an additional control mechanism to respond to environmental cues and thus allow the cell to respond swiftly to appropriate changes in gene expression. This may be particularly important under environmental stress, such as occurring during virus infection. Loss of NCBP3 increased growth of viruses that replicate in the cytoplasm. The exact mechanisms in antiviral defence and the likely involvement in other diseases will be the focus of further studies. Our data show that RNA processing conveys additional complexity that may allow regulatory possibilities for both, cell intrinsic modulation as well as therapeutic intervention.

## Methods

### Cells and reagents

HeLa S3 (CCL-2.2) and Vero E6 cells (CRL-1586) were purchased from ATCC. Human THP-1, NIH3T3 and murine RAW 264.7 macrophages have been described previously^48^. HeLa Kyoto cells stably expressing GFP-tagged human C17orf85/NCBP3, NCBP2 and RAB5C from bacterial artificial chromosomes under control of their endogenous promoter were kindly provided by Ina Poser and Tony Hyman^33^. All cell lines were maintained in DMEM (PAA Laboratories) containing 10% fetal calf serum (PAA Laboratories) and antibiotics (100 U ml^−1^ penicillin and 100 μg ml^−1^ streptomycin). Streptavidin-agarose beads were obtained from Novagen, GFP-Trap-coupled agarose beads were from Chromotek, and γ-Aminohexyl m^7^GTP- and GTP-agarose beads from Jena Bioscience and Biorbyt, respectively. Primary antibodies (1:1000 dilution for western blotting if not stated otherwise) used in this study were as follows: C17orf85 (Atlas Antibodies; HPA008959), NCBP1, SRRT, EIF4A3 and ALYREF (Thermo Scientific; PA 5–30098, PA5–31593, PA5–30329 and MA 1–26754), THOC5, PHAX and KPNA3 (Novus Biologicals; NBP1–19160, NBP2–22268 and NB100–81650), GFP (Invitrogen; A6455), PABP1 and β-tubulin (1:500 dilution for western blotting) (Santa Cruz; 10E10, sc-9104), histone H3 (Abcam; ab1791–100) and EIF4E (Cell Signaling; C46H6). Antibodies against NCBP2 (1:500 dilution for western blotting) were raised by immunizing rabbits with recombinant full-length protein purified from *E. coli*. Antibodies against β-actin (Santa Cruz; sc-47778), His-tag (Santa Cruz; sc-8036) and secondary antibodies detecting mouse or rabbit IgG (Jackson ImmunoResearch) were horseradish peroxidase-coupled. 4′,6-diamidino-2-phenylindole (DAPI) and secondary antibodies used for immunofluorescence were purchased from Invitrogen. Vesicular stomatitis virus M2 (mutant VSV with the M51R substitution in the matrix protein), SFV and EMCV have been described previously^49^.

### RNAi-mediated knockdown

Duplex siRNAs were transfected using either siPrime transfection reagent (GE Healthcare) or the Neon Transfection System (Invitrogen) for target gene knockdowns. Transfection was performed according to the manufacturer's instructions for HeLa or NIH3T3 cells. Briefly, we transfected 200 pmol of siRNA per 1 × 10^6^ cells and repeated transfection 48 h later under the same conditions. For experiments where two genes were silenced simultaneously, we added scrambled-siRNA to single-gene knockdown controls. Cells were analysed at the indicated time points after the second transfection. Duplex siRNAs were either purchased from Qiagen or synthesized by the Core Facility at the MPI of Biochemistry. siRNA target sequences were as follows: human NCBP3 (#1: 5′-AAGAGCCGGTTAGATAACTTA-3′, #2: 5′-TCAGCGGGACGTGATCAAGAA-3′, #3: 5′-ATGACTATGTATGCTGACGAA-3′, #4: 5′-CAGATTGAAGTTAGTCGGGAA-3′), mouse NCBP3 (#1: 5′-TCAGATGTACATAGTAGGCTA-3′, #2: 5′-TCGCTTAGGATCTACACCCAA-3′, #3: 5′-TGGGTTGATGTTGAACAATTT-3′, #4: 5′-CAGATTGAAGTCAGCCGGGAA-3′), human NCBP1 (#1: 5′-CCACAGATGATTGCTGTACTA-3′, #2: 5′-CAGGAACGGCACATCCTAAGA-3′, #3: 5′-CAGGTATGGACTGCTGATAAA-3′, #4: 5′-AGCCGTGTATTTGGTCCGTTT-3′), mouse NCBP1 (#1: 5′-AUGCAGAAAUGGACCGAAU-3′, #2: 5′-CGUCUGGACACGAUGAGUA-3′, #3: 5′-GGUACGAUGUGAAACGGAU-3′, #4: 5′-AGGCCUAACACUCGCGUUU-3′), human NCBP2 (#1: 5′-GCCAUGCGGUACAUAAAUG-3′, #2: 5′-UGGAUGAACUUAUCGUUAA-3′, #3: 5′-GCAUGAGAUAGCCUAAUAA-3′, #4: 5′-AUGAGUAUCGGCAGGACUA-3′), mouse NCBP2 (#1: 5′-CAGCAAAAGUGGUGAUAUA-3′, #2: 5′-GCAAUGCGGUACAUAAACG-3′, #3: 5′-GUAUGGACGUGGACGGUCU-3′, #4: 5′-ACGAGUAUCGGGAGGACUA-3′), huNXF1 (#1: 5′-GAACACGATGATGAACGCGTT-3′, #2: 5′-GATGACATGTCTAGCATTGTT-3′), GFP (5′-AAGCAGCACGACUUCUUCAAG-3′) and scrambled (5′-AAGGTAATTGCGCGTGCAACT-3′).

### Cell growth assays

For cell growth assays, 1 × 10^4^ cells were seeded in 24-well dishes and cell titres determined after the indicated time points using CellTiter-Glo (Promega) according to the manufacturer's instructions with the following modifications: cells were washed once with 1 × PBS and incubated with 100 μl CellTiter-Glo reagent (diluted 1:5 in 1 × PBS) for 10 min. Luminescence was measured using an Infinite 200 PRO series microplate reader (Tecan).

### Immunofluorescence and RNA-FISH

For immunofluorescence, HeLa cells grown on coverslips were fixed with 4% (w/v) paraformaldehyde for 10 min, permeabilized with 0.1% (v/v) Triton X-100 for 10 min and washed three times with blocking buffer (1 × PBS containing 0.1% fetal calf serum (v/v)). Cells were then incubated with primary antibody (1:500 dilution), followed by incubation with secondary antibodies (1:200 dilution) covalently linked to fluorophores and DAPI. Coverslips were mounted on microscope slides using ProLong Gold Antifade Reagent (Molecular Probes). Confocal imaging was performed using a LSM780 confocal laser scanning microscope (Zeiss, Jena, Germany) equipped with a Plan-APO × 63/numerical aperture 1.46 oil immersion objective (Zeiss).

For RNA-FISH analysis, cells grown on coverslips were fixed with 4% (v/v) PFA for 10 min, followed by 100% (v/v) ethanol for 10 min and washed in 70% (v/v) ethanol. Subsequently, cells were incubated with 1 M Tris-HCl pH 8 for 10 min, followed by incubation with hybridization buffer (2 ng μl^−1^ 5′-Cy5-labelled oligo (dT)_45_ (Sigma), 0.5 μg μl^−1^ transfer RNA (Ambion), 1% (v/v) bovine serum albumin, 10% (v/v) dextran sulphate, 20% (v/v) deionized formamide and 2 × sodium saline citrate (SSC)) for 3 h at 30 °C in a humid chamber. After washing once with 4 × SSC and twice with 2 × SSC, cells were incubated with DAPI diluted in 2 × SSC containing 0.1% (v/v) Triton X-100. Finally, cells were washed twice with 2 × SSC and coverslips placed on a microscope slide. Confocal analysis was performed as described above. Quantification of area and intensity of cytoplasm versus nuclei was performed with Volocity 6.3 analysis software (PerkinElmer, Waltham, MA, USA). With the protocol four populations of objects were defined: (1) Nuclei were masked using a DAPI channel with a threshold range of 13–100% and restriction to a minimum object size of 50 μm^2^. ‘Fill holes in objects' was applied to remove holes in the mask; (2) cytoplasm including nuclei was masked using a Cy5 channel with an intensity-based threshold of 6–255 and restriction to a minimum object size of 100 μm^2^. (3) A mask for measurements in the cytoplasm was created using ‘Exclusively combine' of the first two masks. (4) Finally, a mask for measurements in the Cy5-stained nuclei was created by subtracting (3) from (2). Within the mask (3) and (4), area and summarized intensity were measured and intensities were normalized to area. The nuclear to cytoplasmic ratio was calculated using the intensities from a minimum of 125 cells.

### Generation of synthetic and *in vitro* transcribed RNA

Synthetic oligoribonucleotides (Chemgenes Corporation) with a 3′-terminal amino linker harbouring either an N7-methylated cap structure (CAP) or a 5′-hydroxyl group (OH) were generated as described previously^19^. RNA oligomers were modified at the 3′ end either with biotin using biotin-*N*-hydroxysuccinimide ester (Epicentre) or fluorophore using DyLight 488 NHS ester according to the manufacturer's instructions and purified by reverse-phase-HPLC. Biotinylated 7SK-as RNA bearing a 5′-CAP or –OH group, respectively, were synthesized by *in vitro* transcription and enzymatic modification of the 5′ termini^19^ and purified using the NucleoSpin RNA II kit (Macherey-Nagel).

### Affinity purifications

For APs with biotin-labelled RNA, streptavidin-affinity resin was first incubated with RNA in TAP buffer (50 mM Tris pH 7.5, 100 mM NaCl, 5% (v/v) glycerol, 0.2% (v/v) Nonidet-P40, 1.5 mM MgCl_2_ and protease inhibitor cocktail (EDTA-free, cOmplete; Roche)) in the presence of 40 U RNase inhibitor (Fermentas) for 60 min at 4 °C on a rotary wheel and excess RNA removed by three washes with TAP buffer. Cell lysates were prepared by snap-freezing cells in liquid nitrogen, incubation in TAP buffer for 30 min on ice and clarification of the lysate by centrifugation at 16,000*g*. RNA-coated beads were incubated with 2 mg of clarified lysate for 60 min at 4 °C, washed three times with TAP buffer, boiled in Laemmli buffer for 10 min at 95 °C and subjected to SDS–polyacrylamide gel electrophoresis and western blot analysis. Uncropped western blots are provided in Supplementary Fig. 8.

To detect proteins binding to m^7^GTP- and GTP-coupled beads, cell lysate was sonicated using a Branson Sonifier 250 with 15 pulses at a duty cycle of 50% and an output control of 1. The lysate was further incubated on ice for 15 min and clarified by centrifugation at 16,000*g* for 10 min at 4 °C. Similarly, we used GFP-Trap beads (Chromotek) for affinity purification of proteins binding to GFP-tagged baits. The sonicated lysate was further treated with 10 U of Benzonase (Core facility, MPI-B) for 30 min on ice before clarification of the lysate.

For co-precipitation of proteins expressed in *E. coli* expression of recombinant proteins was induced overnight at 18 °C in *E. coli* strain BL21-AI using 1 mM isopropyl-β-D-thiogalactoside (Thermo) and 0.2% L-(+)-Arabinose (Santa Cruz). Cells were lysed in HIS buffer (50 mM HEPES pH 7.5, 500 mM NaCl, 5% glycerol and 20 mM imidazole), including 50 U ml^−1^ of Benzonase (Core Facility, MPI-B) and protease inhibitor cocktail (EDTA-free, cOmplete; Roche) using sonication and lysates clarified by centrifugation at 16,000*g* for 10 min at 4 °C. Lysates were used for AP using Ni-NTA spin columns according to the manufacturer's instructions (Qiagen). Ni-NTA-bound proteins were eluted using HIS buffer containing 500 mM imidazole. Eluates were boiled in Laemmli buffer for 10 min at 95 °C and subjected to SDS–polyacrylamide gel electrophoresis and western blot analysis.

### Quantitative LC–MS/MS-based proteomics and bioinformatics

To detect and quantify RNA-binding proteins and proteins bound to GFP-tagged baits by AP and MS, samples were prepared as described above. After the final three washes in TAP buffer, samples were, in addition, washed twice with TAP buffer lacking Nonidet-P40 to remove residual detergent. Three independent APs were performed for each bait. Sample preparations and LC–MS/MS analysis was performed as described previously^19^. Briefly, samples were sequentially digested with LysC (Wako Chemicals USA) and trypsin (Promega), acidified with 0.1% TFA, desalted with C18 stage tips and analysed by liquid chromatography coupled to MS either on Orbitrap XL or Q Exactive instruments (Thermo Fisher Scientific). For analysis of interaction proteomics data, MS raw files were processed with MaxQuant software versions 1.4.1.8 and 1.4.2.3 (ref. 50) using the built-in Andromeda engine to search against human and mouse proteomes (UniprotKB, release 2012_06) containing forward and reverse sequences. In MaxQuant, the label-free quantification (LFQ)^51^ algorithm and Match Between Runs option were used. Only proteins identified on the basis of at least two peptides and a minimum of three quantification events in at least one experimental group were considered. LFQ protein intensity values were log-transformed and missing values filled by imputation. Specific enrichment was determined by multiple equal variance *t*-tests with permutation-based false discovery rate (FDR) statistics, performing 250 permutations. FDR thresholds and *S*_0_ parameters were empirically set to separate background from specifically enriched proteins.

For total proteome analysis, HeLa cells were electroporated twice with siScrambled or siRNAs against NCBP1, NCBP2, NCBP3 or NCBP2 and NCBP3 and lysed 5 days after knockdown. Three knockdowns were performed in parallel. Cells were pulsed with SILAC Arg10- and Lys8-containing medium for the last 6 h (ref. 49). Cells were lysed in SDS lysis buffer (50 mM Tris pH 7.5, 4% SDS (v/v)), boiled for 5 min at 95 °C and sonicated. Fifty-microgram aliquots were reduced with 10 mM dithiothreitol, alkylated with 55 mM iodoacetamide and precipitated with 80% acetone (v/v) at −20 °C overnight. Pellets were dissolved in 6 M urea—2 M thiourea, digested with LysC and trypsin and desalted peptides analysed by LC–MS/MS on a Q Exactive instrument. Raw MS data were processed with MaxQuant 1.5.1.6 using the LFQ and iBAQ algorithms and the Match Between Runs option only considering light labelled amino acids. LFQ intensities were log-transformed and left non-imputed.

For functional annotation analysis, we used DAVID^52^ (database for annotation, visualization and integrated discovery; http://david.abcc.ncifcrf.gov). Proteomics data were analysed using Perseus, results were plotted using R (www.R-project.org) and GraphPad Prism version 5.02 and visually adapted using Adobe Illustrator.

### RNA immunoprecipitation sequencing

HeLa cells expressing GFP-tagged human NCBP3, NCBP2 or RAB5C were pelleted by centrifugation at 500*g* for 10 min at 4 °C and washed twice with ice-cold PBS. Cells were lysed in an equal volume of RIP lysis buffer (10 mM HEPES pH 7.0, 100 mM KCl, 5 mM MgCl_2_, 25 mM EDTA, 0.5% (v/v) Nonidet-P40, 1 mM dithiothreitol, protease inhibitor cocktail (EDTA-free, cOmplete; Roche)) for 30 min on ice in the presence of 100 U ml^−1^ RNase inhibitor (Fermentas) and lysates clarified by centrifugation at 9,000*g* and 4 °C for 10 min. Clarified lysates were incubated with GFP-Trap beads (Chromotek) in RIP wash buffer (50 mM Tris pH 7.4, 150 mM NaCl, 1 mM MgCl_2_, 0.05% (v/v) Nonidet-P40) containing 25 mM EDTA, protease inhibitors and 100 U ml^−1^ RNase inhibitor for 4 h at 4 °C on a rotary wheel, followed by five washes with RIP wash buffer. Beads were then resuspended in Trizol (Invitrogen) and RNA isolated using the Direct-zol RNA MiniPrep kit (Zymo Research) according to the manufacturer's instructions. Three RNA immunoprecipitations per bait were carried out in parallel. RNA quality was assessed on a Genetic Analyzer (Agilent) and TruSeq RNA library construction and next-generation sequencing were performed by the Max Planck-Genome-Center Cologne, Germany (http://mpgc.mpipz.mpg.de/home/). All samples were sequenced on an Illumina HiSeq2500 platform at 15 million 100-bp single reads per sample. After quality control of the sequencing libraries, reads were trimmed and mapped against the Ensembl genome annotation and the human genome assembly (hg19/GRCh37) using Tophat2 (ref. 53). Reads mapping to ribosomal RNAs or the mitochondrial genome were removed. RNAs binding to NCBP3 or NCBP2 were identified by differential quantification (bait over control) against the Ensembl genome annotation using cuffdiff from the cufflinks package^54^. RNAs with fold changes >2, FDR corrected *P* values <0.01 and minimal read counts of 10 were considered as enriched. To discover preferences of NCBP3 and NCBP2 for different RNA species, we extracted RNA types and gene-model-related features from the Ensembl annotations and plotted them using custom scripts.

### Quantitative RT–PCR

RNA was reverse transcribed using the RevertAid H Minus First Strand cDNA Synthesis kit (Fermentas) and quantified by quantitative RT–PCR using the QuantiFast SYBR Green RT-PCR kit (Qiagen) and a CFX96 Touch Real-Time PCR Detection System (Bio-Rad). Each cycle consisted of 10 s at 95 °C and 30 s at 60 °C, followed by melting curve analysis. Primer sequences were as follows: huMYC (5′-CAGTGGGCTGTGAGGAGGTT-3′ and 5′-CAGGCTCCTGGCAAAAGGT-3′), huSLC43A3 (5′-CATGACATTCCCCACTGGCT-3′ and 5′-GATTCCCCCAATGGTGAGCA-3′), huRNU1-1 (5′-ACTTACCTGGCAGGGGAGATAC-3′ and 5′-ACATCCGGAGTGCAATGGATAA-3′), huRNU4-1 (5′-GCGCAGTGGCAGTATCGTAG-3′ and 5′-GGCGGGGTATTGGGAAA-3′), huNCBP1 (5′-GGCTGCAGCAGATCTTCCTA-3′ and 5′-TCTCCAGGGTCACCATGTACT-3′), huNCBP2 (5′-TTTCCAACATATAACGTACAGCTTTT-3′ and 5′-CTACGTGGAGCTGAGCCAGT-3′), huNCBP3 (5′-GCAGGAAGACAGTTCAGATG-3′ and 5′-ACTTCTTCTGGCTGCTCCAA-3′).

### Homology searches and homology modelling

Orthologous sequences were collected using the web-server morFeus^55^ (http://chimborazo.biochem.mpg.de/morfeus). The multiple sequence alignment was generated using mafft^56^ and submitted to the Gblocks web-server^57^ to select conserved blocks for further phylogenetic analysis. The phylogenetic tree was calculated with Phyml^58^ using standard parameters and bootstrapping with 100 iterations. The resulting tree was displayed using Dendroscope^59^ and visually adapted using Adobe Illustrator.

A structural model of the RRM domain of C17orf85/NCBP3 (UniProt entry Q53F19) was obtained using the HHpred server^60^ for the detection of homologous structures, and subsequent comparative modelling using Modeller^26^ (as integrated in the Bioinformatics Toolkit (http://toolkit.tuebingen.mpg.de)) with the RRM of human poly(A) adenylate-binding protein 1 (PDB code 4f02) as a template model. The final best model was chosen according to DOPE (Descrete Optimized Protein Energy) scores^26^. Structure analysis and electrostatic surface potential calculations were done in Pymol (The PyMOL Molecular Graphics System, Version 1.5.0.4, Schrödinger, LLC).

### Nuclear/cytoplasmic fractionation of mammalian cells

Cells grown in a 10-cm dish were fractionated as described previously^61^ with the following modifications: RNLa buffer was replaced by C/N buffer (10 mM Tris-HCl pH 7.5, 140 mM NaCl, 1.5 mM MgCl_2_, 10 mM EDTA and 0.5% (v/v) NP-40) containing protease inhibitor (EDTA-free, cOmplete, Roche) and Benzonase Nuclease (Novagen). For western blot analysis, we loaded 4% of the total cell lysate and 20% of the cytoplasmic and nuclear fractions.

### Cloning and expression of recombinant proteins

The long isoform of C17orf85/NCBP3 (GenBank NM_001114118.2) was cloned from human complementary DNA (cDNA) into vector pDONR221 (Invitrogen). Mutations and truncations in C17orf85/NCBP3 were introduced by site-directed mutagenesis and PCR, respectively. Expression constructs containing N-terminal His-tagged full-length C17orf85/NCBP3 (pETG10A-NCBP3) or its mutant versions (pETG10A-NCBP3-D134A and -ALAA) were generated using the Gateway system (Invitrogen) as described earlier^49^. Expression constructs for N-terminal MBP/His-tagged full-length NCBP3 and NCBP3 expressing amino acids 1–282, as well as SII-tagged NCBP1 were generated by SLIC cloning as described elsewhere^62^. pETM44 expressing MBP/His was a kind gift from the Core Facility at the MPI of Biochemistry. Sequences of all cloning primers are available on request. Expression of recombinant proteins was induced overnight at 18 °C in *E. coli* strain BL21-AI using 1 mM isopropyl-β-D-thiogalactoside (Thermo) and 0.2% L-(+)-Arabinose (Santa Cruz). Cells were lysed in BL buffer (50 mM Tris-HCl pH 7.5, 500 mM NaCl, 5% glycerol, 5 mM β-mercaptoethanol, 20 mM imidazole and protease inhibitor cocktail (EDTA-free, cOmplete; Roche)) and the cleared lysate was used for further experiments or AP using a HisTrap HP column (GE Healthcare; 17-5247-01). Recombinant untagged wild-type and mutant (ALAA) NCBP3 1–282aa used for MST and CD measurements was purified by the Core Facility at the MPI of Biochemistry. Identity of recombinant NCBP3 was confirmed by MS.

### Affinity measurements

For MST affinity measurements, labelled RNA oligos (CAP0- and OH-RNA-DyLight) were diluted in MST-1 buffer (50 mM Tris-HCL pH 7.4, 150 mM NaCl, 10 mM MgCl_2_, 0.05% (v/v) Tween-20) to a final concentration of 50 nM. A twofold dilution series (16 samples) of NCBP3 1–282aa starting from 204 μM were performed in MST-2 buffer (20 mM Tris-HCL pH 7.4, 300 mM NaCl, 5% (v/v) glycerol). The two components were mixed in equal volumes and the mixture (∼8 μl) was then measured in the Monolith NT.015T at room temperature using a blue light-emitting diode (LED). The MST settings were: LED power (20%), MST laser power (80%), fluorescence before (5 s), MST on (30 s) and fluorescence after (5 s). The normalized fluorescence signal (*F*_norm_=*F*_hot_/*F*_cold_; hot=34.5 s and cold=5.5 s) was analysed and plotted by Monolith NT.015T analysis software. Three individual measurements were combined to calculate the *K*_d_ values with s.e.

### CD spectroscopy

Far ultraviolet CD spectra of wild-type and mutant versions of NCBP3 were recorded on a Jasco J-810 CD-Photometer at room temperature in 20 mM sodium phosphate buffer pH 7.4 and 50 mM NaF. For each sample and the buffer (baseline), four scans were recorded and averaged. The averaged baseline spectrum was subtracted from the averaged sample spectra and the resulting spectra were smoothed using an FFT (Fast Fourier Transform) filter (as part of the software package). Measurements were only made down to wavelengths where the instrument dynode voltage indicated the detector was still in its linear range (190 nm). Spectra are shown as the mean residue elipticity. Secondary structure compositions were estimated using the CONTINLL program^63^.

### Virus infections and determination of virus titres

To determine the impact of siRNA-mediated knockdown of NCBP3 on virus growth, aliquots of 1 × 10^5^ HeLa cells that had been transfected for 48 h with siRNA targeting NCBP3 or GFP as control were infected with VSV-M2, EMCV or SFV at a multiplicity of infection of 0.01. At 24 h post infection, virus titres in supernatants were quantified by 50% tissue culture-infective dose (TCID50) assays on Vero E6 cells.

## Additional information

**Accession codes:** RIP-seq data sets are available from Gene Expression Omnibus (GEO) at the National Center for Biotechnical Information under the accession code GSE71552.

**How to cite this article:** Gebhardt, A. *et al*. mRNA export through an additional cap-binding complex consisting of NCBP1 and NCBP3. *Nat. Commun*. 6:8192 doi: 10.1038/ncomms9192 (2015).

## Supplementary Material

Supplementary Information Supplementary Figures 1-8 and Supplementary References

Supplementary Data 1 LC-MS/MS identified proteins binding to 5'modified RNAs.

Supplementary Data 2 Abbreviations and accession numbers of NCBP3 orthologous proteins.

Supplementary Data 3 Quantitative proteomics after NCBP knockdown.

Supplementary Data 4 LC-MS/MS identified proteins in NCBP3 and NCBP2 precipitates.

Supplementary Data 5 Data tables for the quantification of transcript binding to NCBP3 and NCBP2 measured by RIP-Seq.
