# Supplementary material for: mRNA export through an additional cap-binding complex consisting of NCBP1 and NCBP3
Source: Nat Commun. 2015 Sep 18;6:8192. doi: 10.1038/ncomms9192 (PMC4595607; doi:10.1038/ncomms9192)
Supplement: Supplementary Information — Supplementary Figures 1-8 and Supplementary References [file ncomms9192-s1.pdf]

Supplementary Figure 1

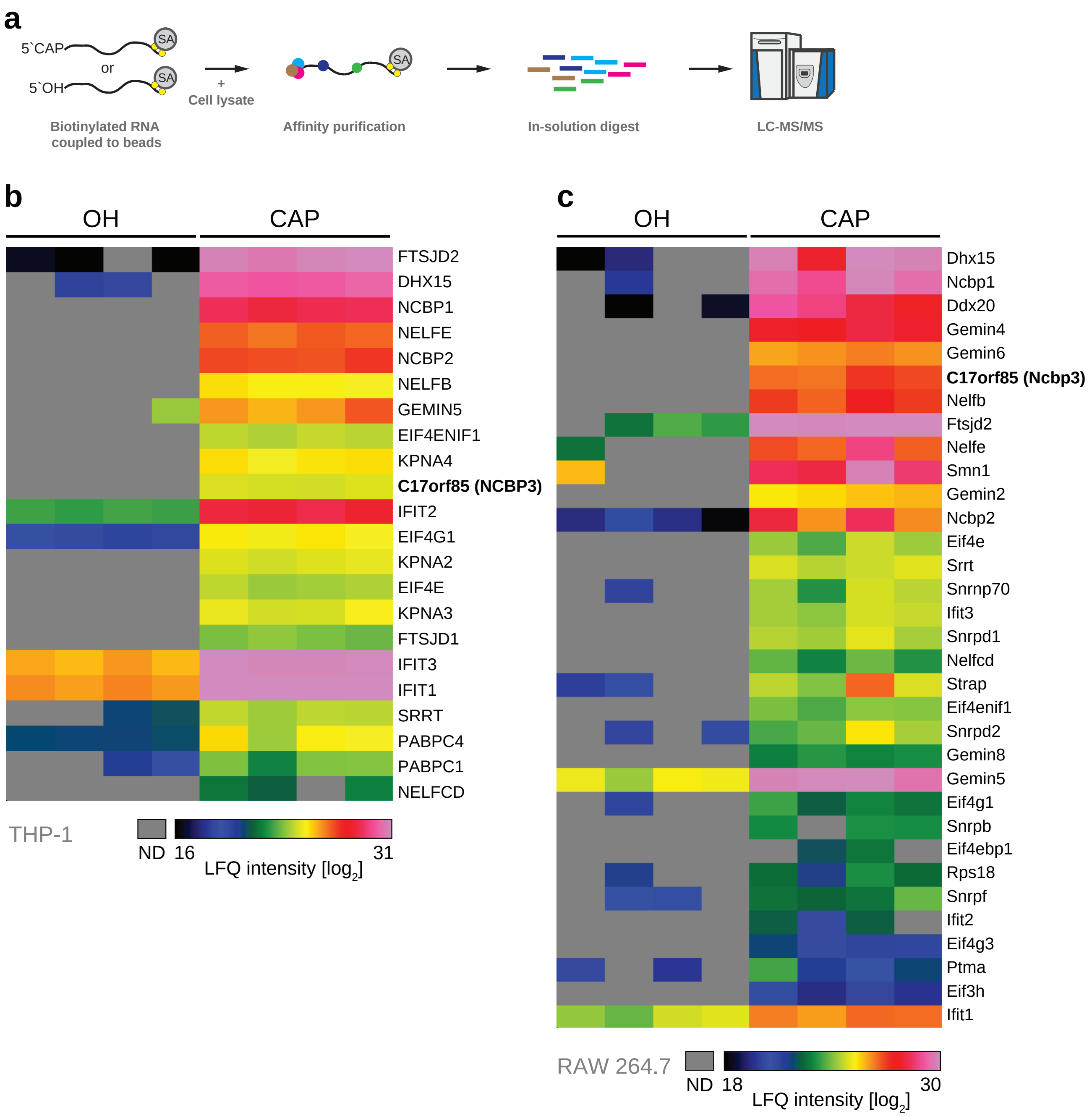

Supplementary Figure 1. Heat map of proteins enriched by capped RNA.
(a) Schematic of quantitative interaction proteomics approach to identify proteins associated with capped RNA. Synthetic biotinylated RNA oligos harbouring either a 5'm^7 guanine cap structure (CAP) or hydroxyl group (OH) were coupled to streptavidin beads, incubated with cell lysates, and bound proteins identified by shotgun liquid chromatography-tandem MS (LC-MS/MS). **(b, c)** Heat maps showing hierarchical clustering (Euclidean distances) of proteins significantly enriched by synthetic cap-RNA oligos (compared to OH-oligos) in murine RAW 264.7 **(b)** and human THP-1 macrophages **(c)**. The plots show non-imputed log2 transformed LFQ intensities for each individual replicate in rainbow colours (see colour scale). Grey colour denotes missing values (ND).

Supplementary Figure 2

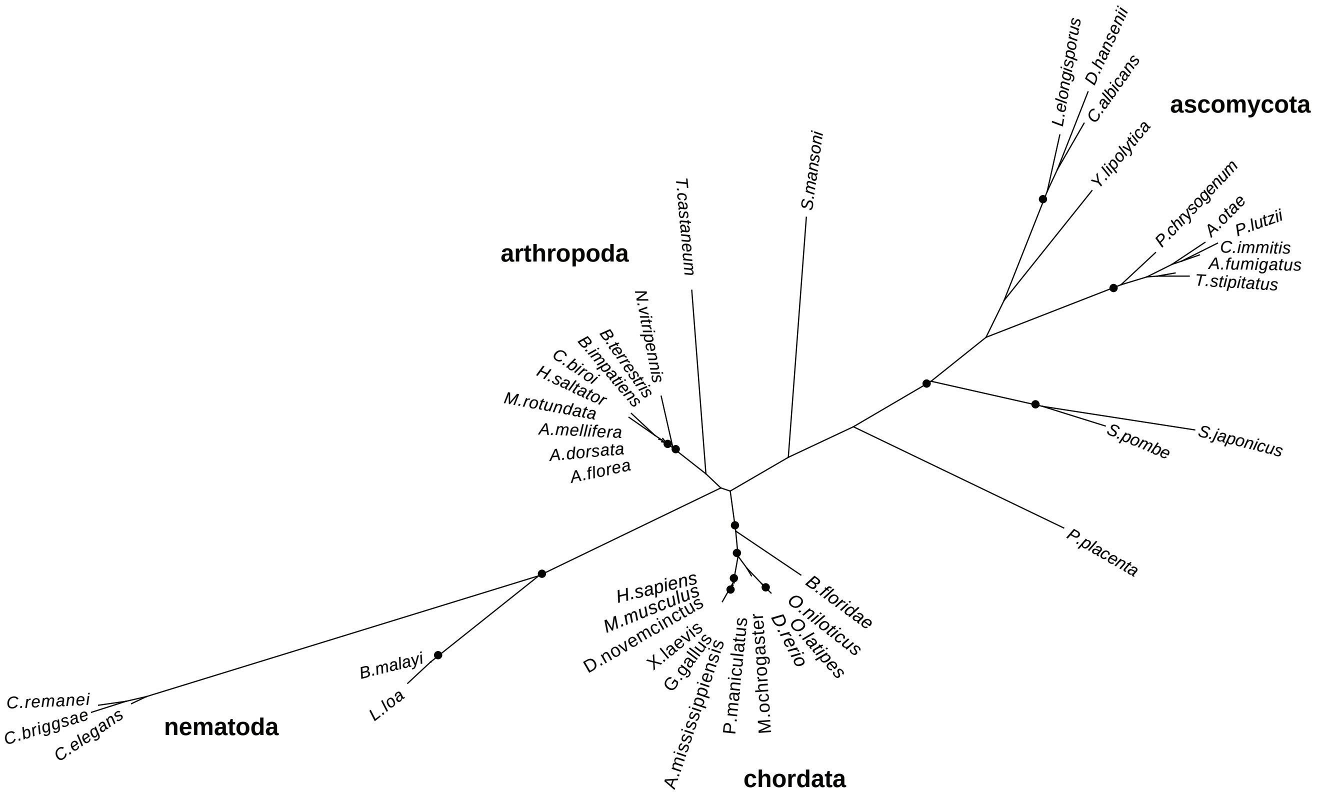

Supplementary Figure 2. NCBP3 is an evolutionary highly conserved protein. Phylogenetic tree of the NCBP3 family of proteins. NCBP3 can be found in Chordata, Arthropoda, Nematoda and Fungi. It is, however, not found in the model organisms Drosophila melanogaster (or any sequenced Muscomorpha) and Saccharomyces cerevisiae (or any sequenced Saccharomycetaceae). Note that we also detected similarity to proteins from plants (green and red algae), which were omitted from further analysis. Dots indicate stable tree branches according to Phyml bootstrapping. Species abbreviations and accession numbers are shown in Table S2.

Supplementary Figure 3

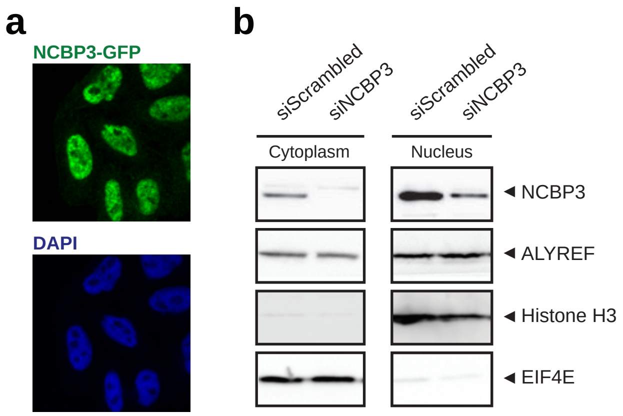

Supplementary Figure 3. Subcellular distribution of NCBP3.
(a) Immunofluorescence analysis to determine the cellular localization of NCBP3. HeLa cells stably expressing NCBP3-GFP were stained with antibodies against GFP (green) and DAPI (blue) and analysed by confocal microscopy. **(b)** Subcellular fractionation of RNAi-treated HeLa cells. Cells were treated with siRNA against NCBP3 or siScrambled as control, and cytoplasmic (C) and nuclear (N) fractions were analysed by western blotting using antibodies against endogenous NCBP3 and ALYREF. Histone H3 and EIF4E were used as controls.

Supplementary Figure 4

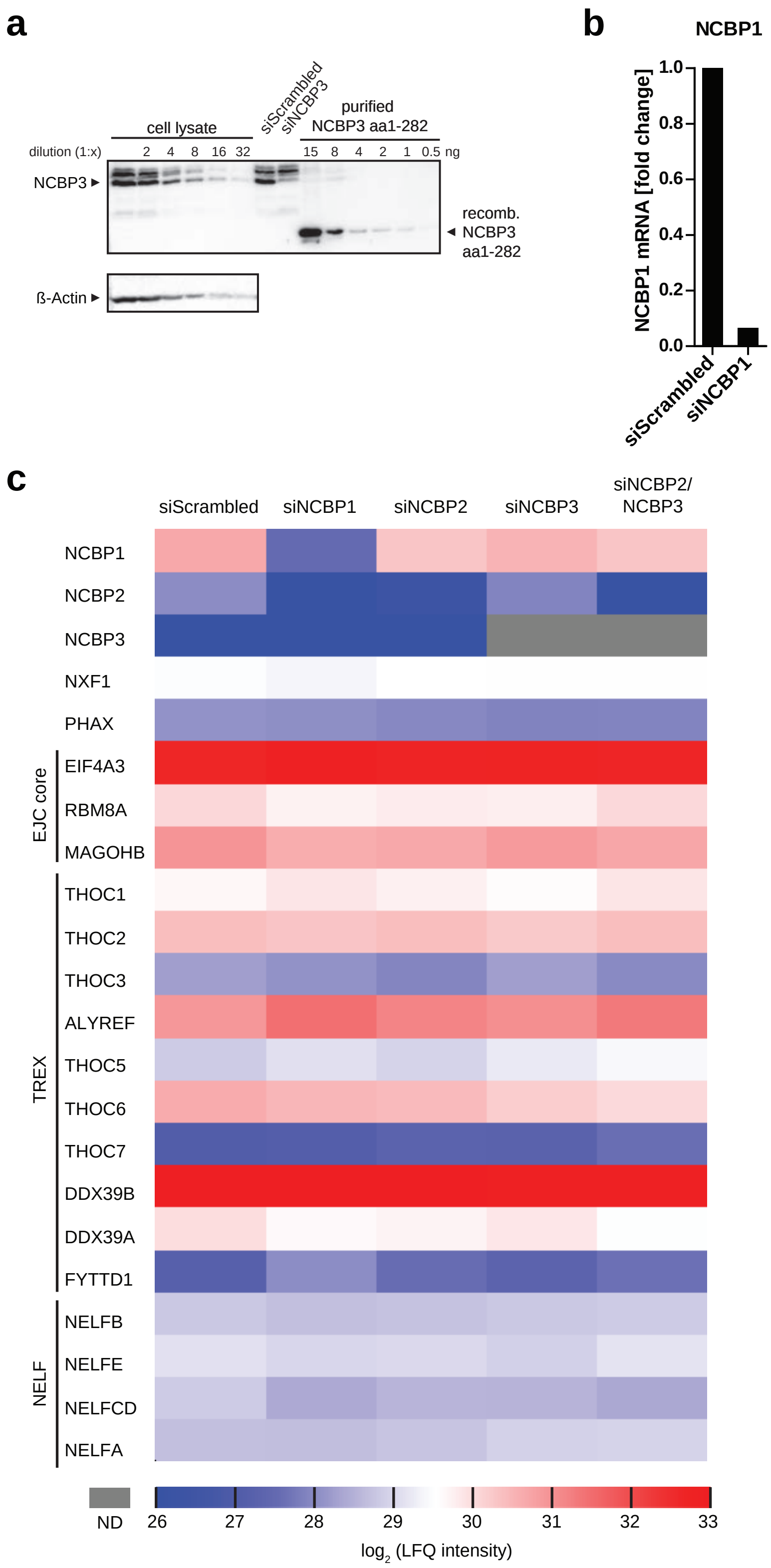

Supplementary Figure 4. Knockdown efficiency of NCBP1, -2 and -3.
(a) Western blot for knockdown efficiency of NCBP3. HeLa cells were treated with siRNAs targeting NCBP3 or control for a total of five days and abundance of NCBP3 tested by western blotting. Serial 2-fold dilutions of HeLa cell lysates and dilutions of purified NCBP3-RRM (1-282) protein were used to assess the sensitivity of the antibody. **(b)** HeLa cells were treated with siRNA against endogenous NCBP1, NCBP2 or NCBP3. To validate knockdown efficiencies. NCBP1, NCBP2 and NCBP3 mRNA levels were determined by qRT-PCR two (siNCBP1) or five days (siNCBP2, siNCBP3) after knockdown. Values were normalized to GAPDH mRNA levels and are expressed as fold change to siScrambled control. **(c)** Proteome analysis of HeLa cells after NCBP knockdowns. HeLa cells were treated with siScrambled control, NCBP1, NCBP2, NCBP3 or NCBP2 and NCBP3 targeting siRNAs for 5 days and analysed by LC-MS/MS. Heat map showing non-imputed log2 transformed averaged LFQ intensities from three replicates for the indicated proteins. Red colour (see scale) represents maximum intensity, blue – minimum. Grey colour indicates

Supplementary Figure 5

a

NCBP3-RRM sequence alignment with PARN-RRM and domain organisation

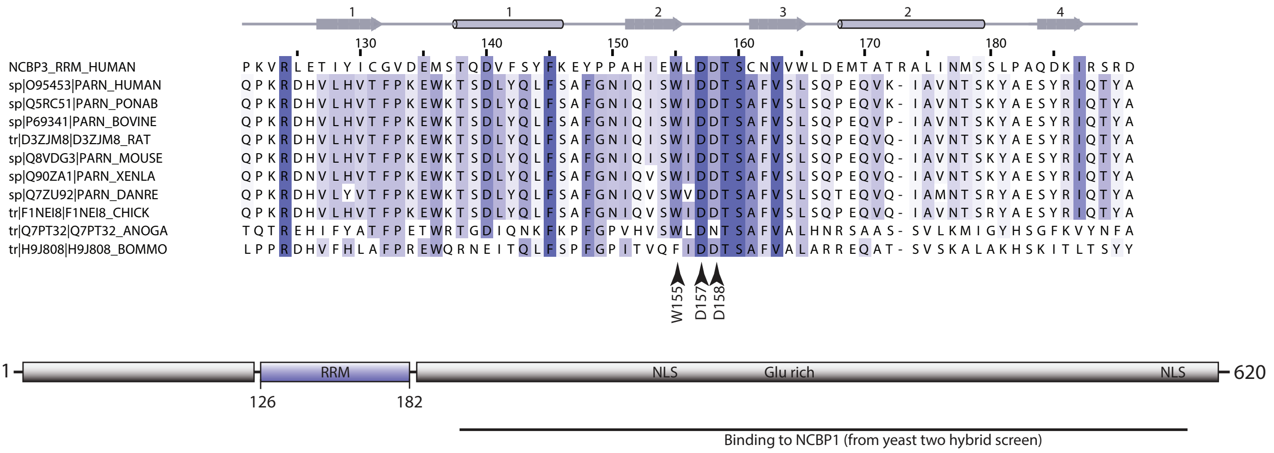

b

NCBP3-RRM sequence alignment with NCBP2-RRM and domain organisation

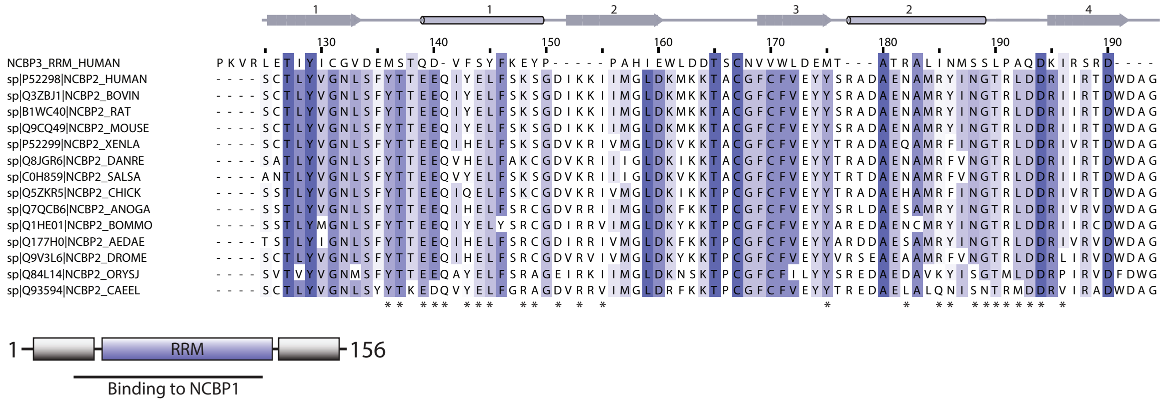

Supplementary Figure 5. NCBP3-RRM amino acid sequence alignment and domain organisation.
Shown are representative sequence alignments (Clustal Omega)[1] between the RRMs of human NCBP3 and Poly(A)-specific ribonuclease (PARN) **(a)**, and between the RRMs of NCBP3 and NCBP2 **(b)**, as well as the respective domain organisations (bar diagrams). Regions that were shown to be necessary for binding to NCBP1 are marked[1, 2]. Sequence numbering refers to full-length human NCBP3. Secondary structure elements are shown above (NCBP3, predicted) and below (NCBP2, PDB code 1h6k10) the alignments. Asterisks in **(b)** mark residues of NCBP2 engaged in the interface formation with NCBP1.

Supplementary Figure 6

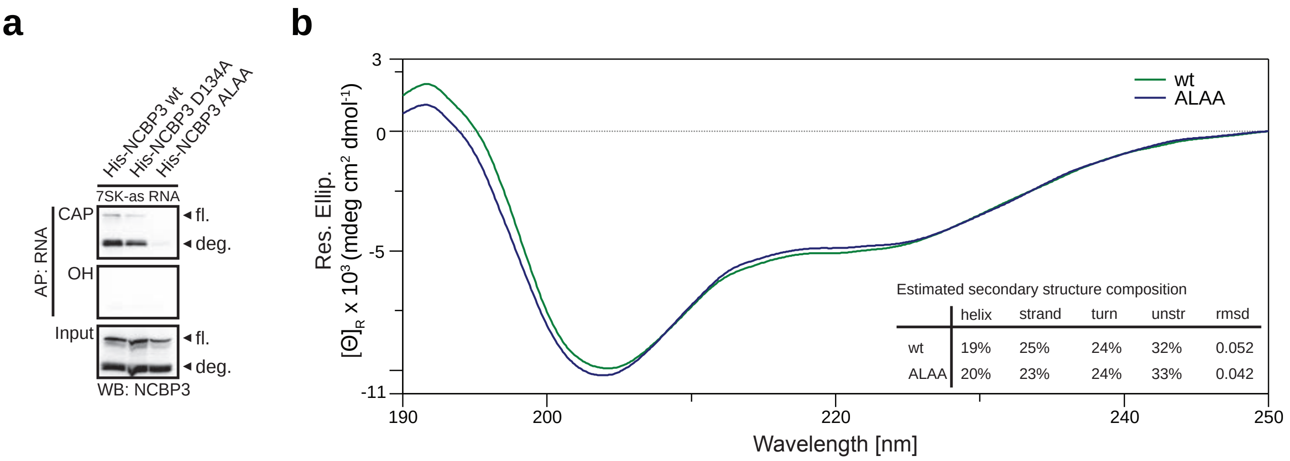

Supplementary Figure 6. Characterization of NCBP3 cap-binding using full-length protein and the RRM-domain.
(a) Binding of recombinant wild-type and mutant NCBP3 to 5’capped RNA. Western blot after affinity purification with biotinylated in-vitro transcribed 7SK-as RNA harbouring either a 5’CAP or OH structure using lysate from E.coli expressing either recombinant full-length wild-type NCBP3 (wt), NCBP3 where aspartic acid at position 134 had been mutated to alanine (D134A), or where tryptophan at position 155 and two aspartic acids at position 157 and 158 had been mutated to alanines (ALAA). Full-length (fl.) recombinant NCBP3 and N-terminal degradation products (deg.) are indicated. **(b)** Far UV circular dichroism (CD) spectra for wild type (wt; green) and mutant (ALAA; blue) NCBP3 (aa 1-282). The overall similarity of the spectra as well as the estimated secondary structure compositions (insert table) are shown.

Supplementary Figure 7

a

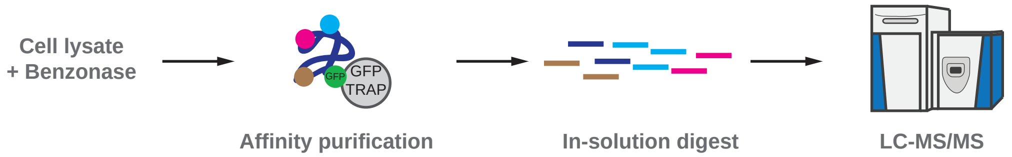

b

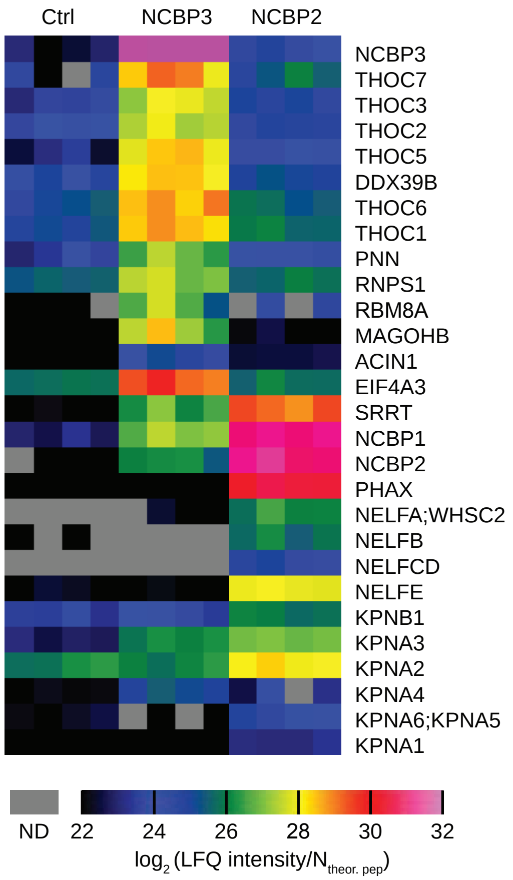

c

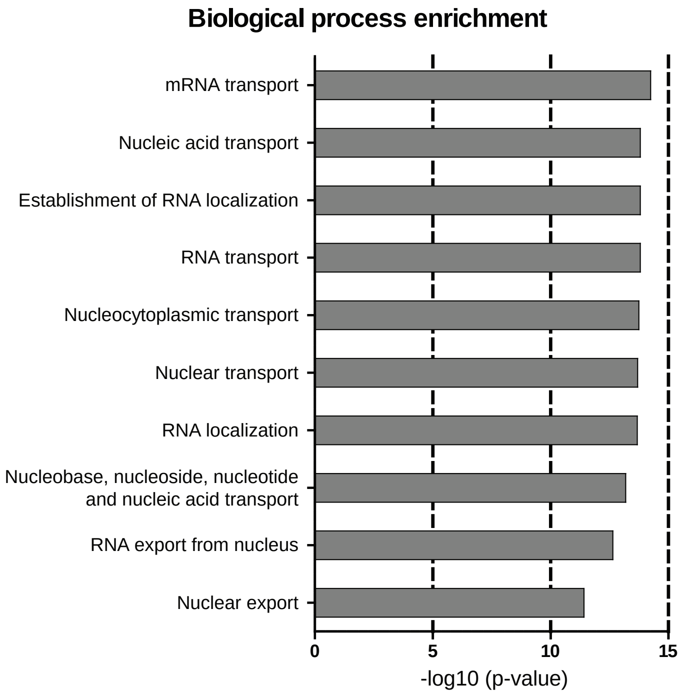

Supplementary Figure 7. Heat map and functional annotation analysis of proteins enriched by NCBP3 and NCBP2.
(a) Schematic of quantitative interaction proteomics approach to identify proteins associated with NCBP3 or NCBP2. GFP-Trap beads were incubated with benzonase treated cell lysates and bound proteins identified by shotgun liquid chromatography-tandem MS (LC-MS/MS). **(b)** Heat map showing hierarchical clustering (Euclidean distances) of proteins significantly enriched by precipitation of GFP-NCBP3 and GFP-NCBP2 as compared to GFP-RAB5C (ctrl) belonging to the TREX complex, NELF complex, EJC, Importins or CBC. The plot shows non-imputed log2 transformed LFQ intensities for each individual replicate in rainbow colours (see colour scale). Grey colour denotes missing values (ND). **(c)** Functional annotation analysis of enriched gene ontology biological processes (GOBP). The gene list of enriched NCBP3 interactors (p-value < 0.001 and LFQ intensity fold change ≥ 8 as compared to GFP-RAB5C) has been analysed for GOBP enrichment using DAVID. Plotted are the top ten GOBP with the lowest p-values.

Supplementary Figure 8

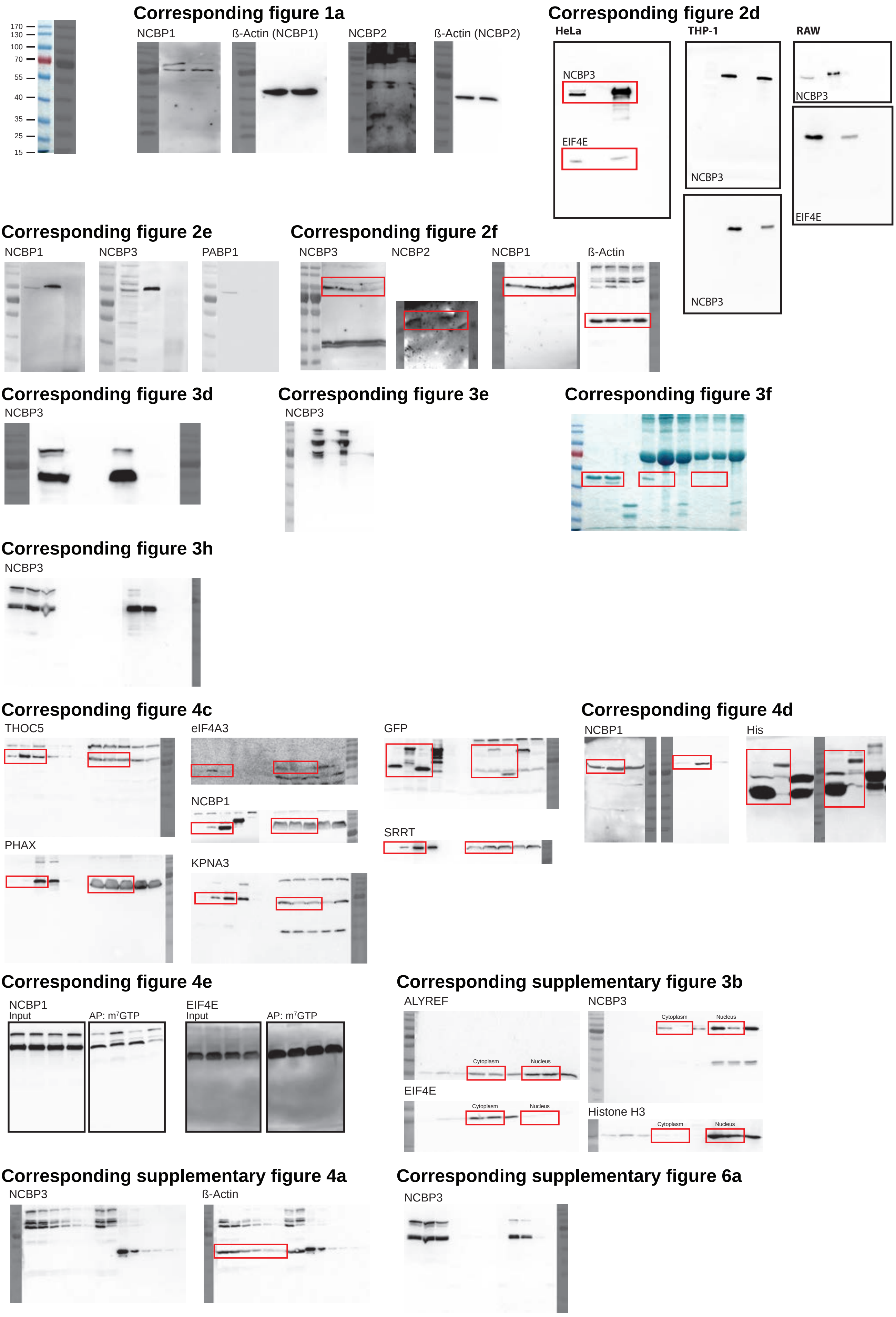

Supplementary Figure 8. Original western blots.

Supplementary References

1. McWilliam, H. *et al.* Analysis Tool Web Services from the EMBL-EBI. *Nucleic acids research* **41**, W597-600 (2013).

2. Mazza, C., Ohno, M., Segref, A., Mattaj, I. W. & Cusack, S. Crystal Structure of the Human Nuclear Cap Binding Complex. *Mol. Cell* **8**, 383–396 (2001).

3. Hegele, A. *et al*. Dynamic protein-protein interaction wiring of the human spliceosome. *Mol. Cell* **45**, 567–80 (2012).
